# Trivalent ions kinetic-gating for producing high-concentration and shelf-stable plasmid DNA/PEI particles

**DOI:** 10.1038/s41467-026-73921-4

**Published:** 2026-06-02

**Authors:** Jinghan Lin, Yizong Hu, Turash H. Pial, Kailei D. Goodier, Di Yu, Paetra Brailsford, Maria Choi-Ali, Jonathan T. Feng, Sixuan Li, Yining Zhu, Jingyao Ma, Leonardo Cheng, Xiaoya Lu, Nicole Korinetz, Marine Guise, Tza-Huei Jeff Wang, Tine Curk, Hai-Quan Mao

**Affiliations:** 1 Institute for NanoBioTechnology, Johns Hopkins University, Baltimore, MD USA; 2 Department of Materials Science and Engineering, Johns Hopkins University, Baltimore, MD USA; 3 Translational Therapeutic and Regenerative Engineering Center, Johns Hopkins University School of Medicine, Baltimore, MD USA; 4 Department of Biomedical Engineering, Johns Hopkins University School of Medicine, Baltimore, MD USA; 5 Biogen Inc., Cambridge, MA USA; 6 Department of Mechanical Engineering, Johns Hopkins University, Baltimore, MD USA; 7 Department of Chemical and Biomolecular Engineering, Johns Hopkins University, Baltimore, MD USA; 8 Sartorius Polyplus, Illkirch-Graffenstaden, France; 9 Present Address: David H. Koch Institute for Integrative Cancer Research, Massachusetts Institute of Technology, Cambridge, MA USA

**Keywords:** Transfection, Biomedical engineering, Nanoparticles, Nanobiotechnology

## Abstract

Scalable, cost-effective manufacturing remains a major barrier to the clinical translation of viral vector–mediated gene therapies. The widely used transfection method for producing adeno-associated virus (AAVs) and lentivirus uses plasmid DNA (pDNA)/polyethyleneimine (PEI) particles loaded with multiple plasmids; however, these particles must be prepared at low concentrations and used immediately, limiting scalability and reproducibility. Here we show a kinetic-gating strategy in which transient binding of trivalent citrate ions slows complexation, modulating charge-neutralization kinetics and delaying particle nucleation, enabling the formation of stable, highly concentrated pDNA/PEI particles. By incorporating citrate, we prevent aggregation and achieve uniform assembly at high concentrations, enabling a ten-fold increase in DNA concentration (to 0.2 mg/mL) and reduced dosing volumes. The method is robust across mixing conditions, compatible with standard manufacturing workflows, and maintains AAV production efficiency across scales. These results establish a simple and generalizable approach to control polyelectrolyte assembly kinetics, improving the scalability and reliability of viral vector production.

## Abstract (web summary)

Scalable manufacturing remains a barrier to viral vector–mediated gene therapies. Here the authors develop a citrate ion-gating strategy to control plasmid/polyethyleneimine assembly, enabling stable, high-concentration transfection particles that improve the scalability, consistency, and efficiency of viral vector production.

## Introduction

Gene therapy has evolved as a transformative modality for treating disease and improving healthcare. In particular, viral-vector gene therapies have moved swiftly from concept to the clinic^1–3^. Current good manufacturing practice (GMP) production of adeno-associated virus (AAV) still relies on the transient transfection of HEK293 derivatives with plasmid DNA (pDNA)/poly(ethyleneimine) (PEI) polyplexes, a workflow first reported nearly three decades ago^4–7^. Despite continuous optimizations^5,8–11^, the field continues to struggle with several technical challenges, such as a lack of understanding of critical properties that govern transfection efficiency^12,13^, batch-to-batch variability^14^, poor stability^15,16^, and complexation behaviors that are inconsistent across different scales and processes^17^.

These limitations manifest as a restrictive concentration ceiling—commercial protocols cannot exceed 20 µg/mL plasmid^18^ before aggregates precipitate. Instability further hampers operations; polyplexes lose colloidal integrity within minutes at ambient temperature and cannot survive freeze-thaw cycles, enforcing a just-in-time workflow. Meanwhile, to tame uncontrolled nucleation and growth, manufacturers rely on multi-step addition schedules or dedicated in-line mixers^19^, which add complexity and a capital burden^20^. Collectively, these challenges increase facility size, labor, and buffer volumes, ultimately constraining the global supply of gene therapy vectors.

Formulation studies have explored buffer additives to moderate PEI–pDNA interactions. Notably, trivalent citrate ions have been used as a formulation excipient to enhance in vitro transfection or to extend bench stability to a few hours. Representative studies include low-molecular-weight PEI–citrate polyplexes that improved luciferase expression in B16-F10 cells^21^, citric-acid-based polyester elastomer as a scaffold for pDNA/PEI complexes^22^, and recent reports that citrate reduces PEI cytotoxicity while conferring 4–24 h colloidal stability^23–25^. These contributions demonstrate the feasibility of introducing multivalent anions, yet they all employed empirically selected citrate concentrations and did not elucidate the underlying rate processes.

Here, we introduce a kinetic-gating strategy in which transient, reversible coordination of trivalent citrate ions slows initial PEI–pDNA complexation to match the slower micromixing time of routine unit operations. This decouples nucleation from growth, yielding uniformly sized polyplex seeds that predictably grow to the target 400–600 nm range at a plasmid concentration up to 200 µg/mL. The resulting particles (i) maintain a high degree of uniformity after 4 h at ambient temperature and after a freeze–thaw cycle, (ii) are produced in a single-buffer step that is robust across different mixing modalities, and (iii) support AAV titers in a 50-L stirred-tank bioreactor that match or exceed an industry benchmark run at Biogen (USA). Supported by coarse-grained molecular dynamics (MD) simulations, isothermal titration calorimetry (ITC), and particle characterization, we demonstrate that citrate acts as a reversible multivalent counterion that attenuates PEI charge density during the critical milliseconds of mixing. This mechanistic insight converts multivalent ions such as citrate from an empirical additive into a design lever for scalable bioprocessing.

## Results

### Kinetic gating represents a paradigm shift from turbulence-driven mixers to ion-mediated rate control

In a standard assembly protocol for pDNA/PEI transfection particles^26^, we discovered that the colloidal size of these transfection particles using PEIpro® (Sartorius Polyplus) is a critical parameter determining their transfection efficiency, with an optimal size in the range of 400–600 nm^19^. We previously developed a stepwise, electrostatic charge-mediated assembly method that enabled the manufacturing of bench-stable (>4 h at room temperature) and shelf-stable (>1 year at –80 °C) 400–600-nm pDNA/PEI particles and successfully benchmarked their performance in producing lentiviral vectors^19^ (Table 1). With specifically designed flow mixing devices and protocols that enabled continuous-flow preparation of pDNA/PEI particles to improve the reproducibility of cell transfection, the assembly method was proven scalable to a commercially relevant scale, producing such transfection particles at a flow rate of 1 L/min and at a pDNA concentration of up to 50 µg/mL^20^. However, the assembly method requires specialized pumping and turbulent mixing equipment, a dedicated particle manufacturing pipeline, and a time-consuming manufacturing process^20^, which are sub-optimal for adoption in large-scale bioreactors. These limitations motivated the development of a new assembly process based on a trivalent ion-mediated kinetic gating mechanism (Table 1).

**Table 1:** Competitive advantages of the kinetic gating method reported in this study

Features	Industry standard protocol	Kinetic assembly (*stepwise method*)	Kinetic gating (*This work*)
Assembly control mechanism	Concentration-dependent	Mixing kinetics-dependent	PEC complexation kinetics-dependent
Stepwise process	Single step	Two-step	Single step
Allowable pDNA concentrations (µg/mL)	10–20	20–50	20–200
Mixer requirement	None	Confined-impinging jet (CIJ) mixer	None
Preparation time	20–40 min	6 h	20–40 min
Stability of transfection particles	Non-stable	Bench stable >4 h Shelf stable >1 year	Bench stable >4 h Shelf stable >1 year
Scale tested	N/A	5 L	90 mL

**Fig. 1:**
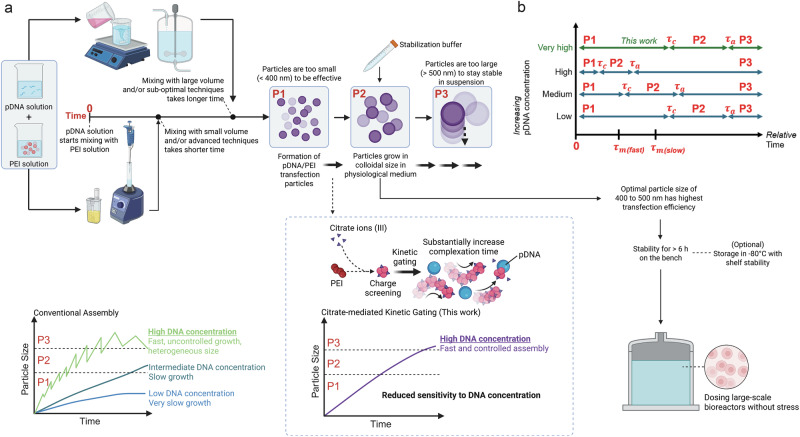
Kinetically controlled assembly process of the shelf-stable pDNA/PEI transfection particles. **a** Schematic overview of the process to prepare pDNA/PEI transfection particles, involving mixing of pDNA cocktail solution and PEI solution that is followed by polyelectrolyte complexation (PECn) and the nanoparticle assembly (NPa) in a medium that bears physiological pH and ionic strength. The size growth of pDNA/PEI particles can be divided into three periods: P1, in which particles possess a size <400 nm; P2, in which particles possess a size between 400 and 600 nm; P3, in which particles possess a size >600 nm. **b** Comparisons of the relative time scales of the mixing, PECn, and NPa steps. The figure was generated by BioRender.com.

The assembly of pDNA/PEI particles with a target size range of 400–600 nm involves two stages (Fig. 1a): the initial polyelectrolyte complexation (PECn) and subsequent nanoparticle assembly (NPa). During PECn, the mixing rate of pDNA solution and PEI solution compared to the complexation rate of the two macromolecules is critical for the ability to generate uniform polyelectrolyte complex (PEC) nanoparticles. The PECn “reaction” is highly energetically favorable; thus, the characteristic complexation time *τ*_*c*_, i.e., the time it takes to complete the complexation between pDNA and PEI, is typically shorter than tens of milliseconds^27^. On the other hand, when pDNA and PEI solutions are mixed using conventional methods, the relatively low and mismatched diffusivities of the two types of macromolecules, due to the large difference in their hydrodynamic size and molecular weight, make homogeneous mixing difficult to attain. Importantly, the theoretical characteristic mixing time *τ*_*m*_, i.e., the time it takes to achieve homogenous mixing of pDNA and PEI components, is two to three orders of magnitude longer than *τ*_*c*_, yielding PEC nanoparticles with a higher degree of size heterogeneity.

One approach to address this challenge is to speed up the mixing rate and mixing efficiency, thus reducing *τ*_*m*_ by reducing the diffusion distance of the two components^28,29^. Previously, we adopted a flash nanocomplexation (FNC) technique^27^ to break the two solution jets into micron-scale sheets through a turbulent mixing regime in a microchamber, drastically reducing the diffusion distance between the two components. Under this condition, all pDNA molecules and PEI molecules complex nearly simultaneously, thereby generating uniform PEC nanoparticles with a high density of residual positive charges that render them colloidally stable. These PEC nanoparticles are then aggregated under controlled conditions to obtain the 400–600 nm transfection particles. However, the implementation of such advanced mixing technology and multi-step procedures may not always be favorable in the development of scale-up transfection processes in bioreactors.

Here, we propose a new trivalent ion-mediated kinetic gating method to substantially slow down the rate of PECn, thus increasing *τ*_*c*_ to match the slower mixing process, thereby improving the uniformity of the PECn. Experimentally, we observe that an engineered citrate-buffered saline (CBS) containing trivalent citrate ions used as the buffer for PECn reduces particle polydispersity and enhances size control across mixing regimes. We hypothesize that citrate functions as a multivalent, reversible chelator of PEI. Due to its small molecular weight and high diffusivity, citrate can rapidly associate with protonated amines on PEI during mixing, transiently reducing the effective positive charge density. This reduction likely decreases the energetic driving force for immediate PEI–pDNA complexation, thereby slowing PEC formation (Fig. 1a). At higher pDNA concentrations, where *τ*_*c*_ is intrinsically shorter, the gating effect becomes more pronounced (Fig. 1b). Given the higher charge density of pDNA, citrate is expected to be displaced during final PEC formation, restoring strong PEI–DNA interactions. We therefore propose that citrate-mediated kinetic modulation enables PEC formation under conditions where *τ*_*c*_ approaches or exceeds *τ*_*m*_, reducing sensitivity to mixing rate and improving uniformity across scales.

Following mixing and the initial PECn process, the surface charges of the pDNA/PEI nanoparticles are drastically reduced due to charge neutralization, and the charge screening conferred by the citrate ions and the high ionic strength in the buffer reduces the barriers of assembly and permits nanoparticles to “aggregate” into larger particles. This NPa process is a relatively slow “reaction,” with a longer characteristic time of *τ*_*a*_, defined as the time it takes to reach the target size of the particles (Fig. 1a), due to the much slower diffusion rate of the nanoparticle units compared with the original pDNA and PEI macromolecules. During this NPa step, we hypothesize that pDNA/PEI particles can form more uniform aggregates at a controlled rate because of the more uniform PECn process mediated by citrate ions. Once the particles grow to the target size, a stabilization buffer dissolved in an acidic pH is used to effectively re-protonate the PEI molecules on the surface of the PEC particles, which can arrest the particle growth and preserve particle uniformity via long-range electrostatic repulsion^19,27^.

### Citrate ion-mediated kinetic gating enhances the uniformity and stability of high-concentration assembly of pDNA/PEI particles

**Fig. 2:**
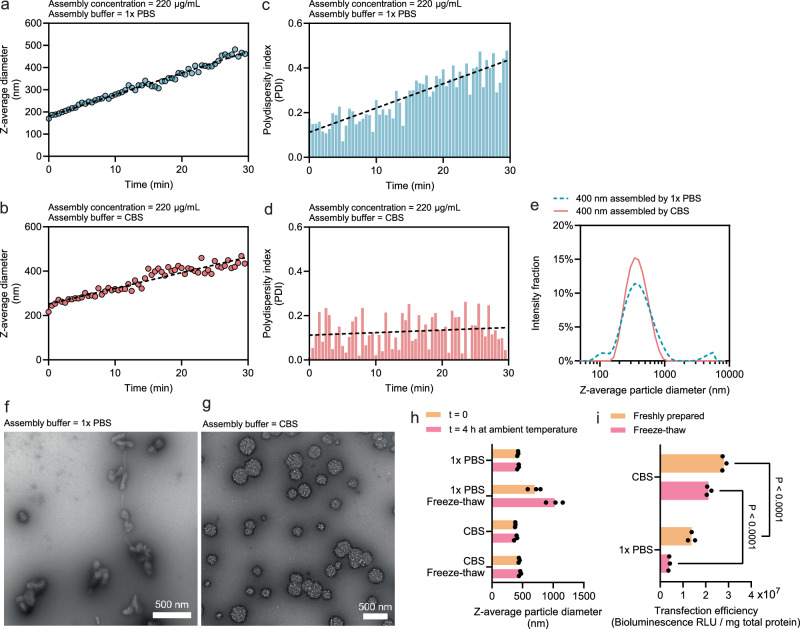
Citrate-mediated assembly of pDNA with PEI generates uniform and stable particles. **a,b** The size growth curve of the assembly of pDNA/PEI particles monitored by consecutive dynamic light scattering (DLS) measurements in the assembly buffer of 1 × PBS (**a**) or citrate-buffered saline, CBS (**b**). **c, d** The polydispersity index (PDI) of the growing pDNA/PEI particles measured for the assembly buffer of 1 × PBS (**c**) or CBS (**d**). **e** The size distribution given by DLS of 400-nm particles assembled by either PBS or CBS buffer. **f, g** Transmission electron microscopy (TEM) assessing the uniformity of 400-nm particles assembled by 1 × PBS (**f**) or CBS (**g**). Similar morphology and relative differences in particle uniformity were observed across three independent experiments. **h, i** The on-bench (ambient temperature) and freeze-thaw stability of 400-nm particles produced by either buffer in terms of the particle size assessed by DLS (**h**), or the transfection efficiency on HEK293T cells (**i**) when the particles were loaded with 10% (w/w) luciferase pDNA. Data are presented as mean (*n* = 3 technical replicates from one particle preparation). Statistical significance was performed using two-way ANOVA followed by Šídák’s multiple comparisons test (two-sided, adjusted for multiple comparisons). Freshly prepared: mean difference = −1.417, 95% CI = −1.661 to −1.172, t = 15.91, adjusted *P* < 0.0001. Freeze–thaw: mean difference = −1.728, 95% CI = −1.972 to −1.484, t = 19.41, adjusted *P* < 0.0001.

In our previous method, the phosphate-buffered saline (PBS, pH 7.0) was used to induce particle growth^19^ by deprotonating nearly 60% of the amine groups on PEI^30^. The salts in the buffer, including Na^+^, K^+^, Cl^-^, and phosphates, provide charge screening to PECn. Here, we modified this buffer to replace a portion of the buffer salts with trisodium citrate, while retaining the same ionic strength, yielding the citrate-buffered saline (CBS). The pH of CBS was tuned to be 7.0. In all the experiments, we targeted a high pDNA concentration and selected the working concentrations of pDNA and PEI solutions to be 440 μg/mL and 580.8 μg/mL, respectively, correlating with a nitrogen-to-phosphate (N/P) ratio of 5.5. In an initial run, we used pipetting as the mixing method to add 100 μL of PEI solution into an equal volume of the pDNA working solution diluted in either PBS or CBS. Continuous DLS measurements revealed that the overall particle growth rates in both PBS and CBS buffers were comparable (Fig. 2a, b), and the average diameter of the particles reached the target size of 400 nm at around 20 min after mixing. However, a sharp difference in the polydispersity index (PDI), a measure of particle uniformity (see definition in “Methods”), was observed. The PDI increased with the average size of the particles assembled in PBS (Fig. 2c), suggesting increasing size heterogeneity as the particles grew; however, the PDI of the pDNA/PEI particles assembled in CBS remained largely unchanged in a range of 0.05–0.2 throughout the entire growth process, which is considered reasonably uniform^31^ (Fig. 2d). The surface charge during assembly was also monitored using zeta potential measurements (Supplementary Fig. 1). At steady state, particles assembled in PBS exhibited a zeta potential of ~40–42 mV, whereas CBS-assembled particles reached a slightly lower plateau of ~35–37 mV, consistently displayed a modest reduction (~ 4–6 mV) in surface charge compared with PBS. This difference in steady-state electrostatic characteristics is consistent with altered ion–polymer interactions during assembly and is further examined in the mechanistic analysis later.

To examine whether the observed kinetic modulation is specific to citrate, we performed additional experiments using Na₂SO₄ (a divalent anion) and sodium EDTA (an alternative multivalent anion) under matched conditions (Supplementary Fig. 2). The divalent sulfate-containing buffer resulted in less controlled particle growth and higher PDIs. In contrast, the tetravalent EDTA exhibited more linear growth and lower PDIs than PBS, qualitatively resembling citrate-mediated behavior. While EDTA supports the generality of the multivalency-mediated effect, citrate remains particularly attractive for translational formulation due to its established biocompatibility and the absence of strong metal-chelation concerns associated with EDTA. These results collectively confirm that multivalent ions influence the kinetics of complex formation and particle growth by temporarily reducing the effective charge density of PEI.

Once the pDNA/PEI particles reach the target size of 400 nm, the particle growth can be arrested by the addition of a stabilization buffer, which contains hydrochloric acid to protonate surface PEI to the maximum degree. Trehalose (19%, w/w) was added to this stabilization buffer to confer stability during cryo-storage. The size stability was verified by DLS, showing a more uniform size distribution of the CBS-assembled particles (Fig. 2e), and by transmission electron microscopy (TEM), examining the morphology of the particles. The pDNA/PEI particles assembled by PBS appeared to be randomly shaped agglomerates with drastically different sizes (Fig. 2f, Supplementary Fig. 3a, b), whereas the particles assembled by CBS were more uniform in size with a predominantly spherical shape (Fig. 2g, Supplementary Fig. 3c, d). Both particles assembled in PBS and CBS buffers remained stable at the ambient temperature for at least 4 h; nonetheless, only particles assembled in CBS remained stable upon a freeze-thaw cycle (Fig. 2h). More importantly, when we encapsulated luciferase pDNA in the particles and transfected HEK293T cells using stabilized particles, 400-nm pDNA/PEI particles assembled in CBS showed a significantly higher transfection efficiency than those prepared in PBS (Fig. 2i), ~2-fold on freshly prepared particles and ~5-fold on freeze-thawed particles, presumably due to the more uniform size distribution within the optimal size range of 400–600 nm.

Of important note, further increasing the PBS concentration, which theoretically increases the degree of charge screening at a higher ionic strength, did not provide a similar benefit to CBS-mediated assembly, but rather resulted in uncontrollable aggregation (Supplementary Fig. 4). We also examined the effect of trisodium citrate concentration in the CBS buffer as multivalent ions on particle supramolecular assembly^32^, the NPa step between *τ*_*c*_ and *τ*_*a*_, that particle growth could not occur when citrate concentration was too low, and uncontrollable aggregation occurs when citrate concentration was too high (Supplementary Fig. 5a). The detailed kinetics analysis of the effects of multi-valent ions on *τ*_*a*_, and the relationship between *τ*_*c*_ and *τ*_*a*_, are beyond the scope of this manuscript and warrants further investigations. Nonetheless, the selected citrate concentration could accommodate a wide range of pDNA concentrations to mediate reproducible particle growth (Supplementary Fig. 5b).

### Molecular dynamics simulation reveals mechanistic insights into the kinetic gating of the pDNA/PEI particle assembly

To explicitly examine the molecular complexation behaviors between pDNA and PEI in PBS and CBS, we conducted molecular dynamics (MD) simulations. We employed a coarse-grained model by condensing 3–4 heavy atoms (e.g., nitrogen, carbon, oxygen, phosphate) into a single particle^33^. This captures the necessary chemical characteristics of the repeating units on each macromolecule, while allowing simulation of sufficiently large length and time scales. The PEI was modeled as a linear polymer chain with 40% of its nitrogen atoms protonated, mimicking physiological conditions^30^.

**Fig. 3:**
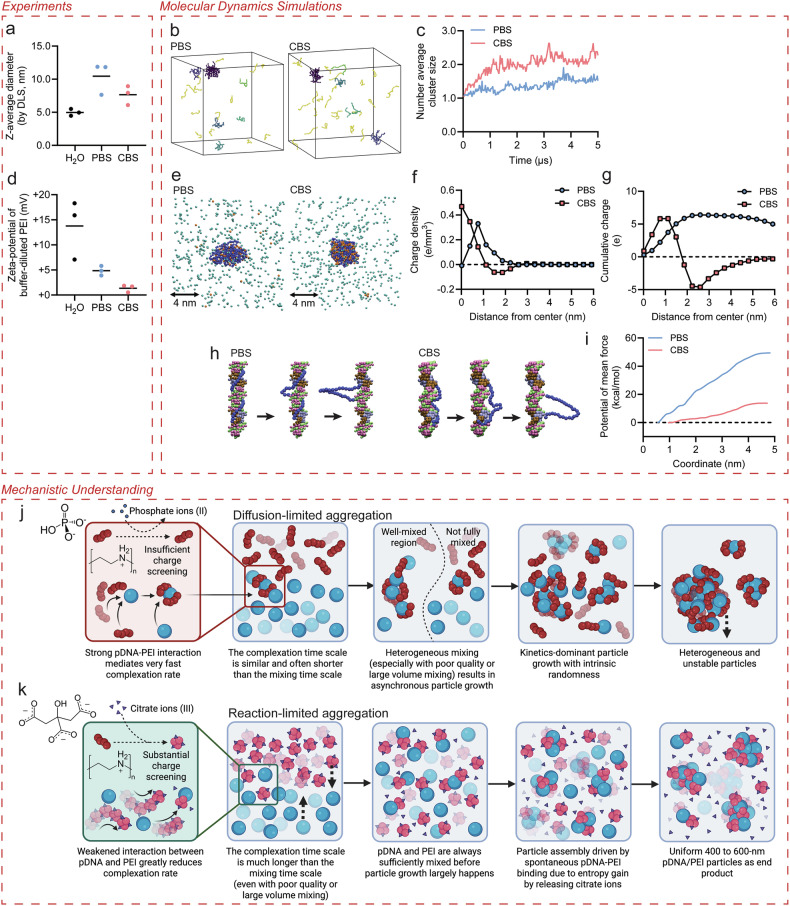
Mechanistic understanding of pDNA/PEI particle assembly mediated by PBS vs. CBS. **a** The size of PEI molecules upon dilution in different buffers. **b, c** MD simulations (**b**) illustrating aggregation behavior of PEI molecules independent of pDNA in different buffers and quantifying the number average cluster size over time (**c**). In (**b**), different color indicates different clusters, yellow indicates unassociated single chains. **d** The zeta-potential of PEI molecules upon dilution in different buffers. **e**–**g** MD simulations (**e**) illustrating ion-PEI binding (Blue: PEI chain; Orange: multi-valent ions; Green: mono-valent ions) and quantifying the charge density (**f**) and the cumulative charge distribution (**g**) from the center of mass of PEI towards the surface. **h**,** i** MD simulations of DNA/PEI complexation in PBS and CBS, showing snapshots of umbrella sampling simulations along different pulling coordinates (**h**) and the free energy of pDNA/PEI binding in different buffers (**i**). **j**, **k** Schematic overview of the hypothetical mechanisms governing pDNA/PEI assembly in PBS (**j**) or CBS (**k**). Data are presented as mean (*n* = 3 technical replicates from one particle preparation). Panels (**j**) and (**k**) were generated with BioRender.

Through DLS, we found that diluting PEI into PBS or CBS only induces a minimal degree of self-assembly of PEI with the counter ions, characterized by a slight increase in the diameter of PEI without further assembly (Fig. 3a). MD simulation showed a similar outcome that at equilibrium, PEI-phosphate interaction and PEI-citrate interaction were apparent (Fig. 3b) without a significant increase in their number-average cluster size over time (Fig. 3c). The fluctuations observed in the number-average cluster size of PEI-citrate shows that the citrate-PEI binding is reversible.

Since complexation is driven by charge, we measured the zeta potential of PEI molecules by phase analysis light scattering (PALS). Substantial charge screening was observed in both CBS and PBS (Fig. 3d), with CBS yielding nearly full charge neutralization. To experimentally quantify the thermodynamic association between buffer ions and PEI, we performed isothermal titration calorimetry (ITC) by titrating CBS or PBS into PEI solution. Citrate-containing injections produced substantially larger heat changes compared to PBS under matched conditions (Supplementary Fig. 6), indicating stronger and more cooperative interactions between citrate and protonated PEI. The integrated heat profile further supports a more significant enthalpic contribution in the citrate system, consistent with multivalent ion pairing and enhanced charge compensation. Due to the multivalent and non-stoichiometric nature of PEI–ion interactions, extraction of a single binding constant is not physically meaningful; the observed heat includes coupled contributions from protonation equilibria and counterion redistribution. Nevertheless, the comparative ITC signatures provide direct thermodynamic evidence that citrate associates more strongly with PEI than phosphate species, supporting the mechanistic interpretation derived from MD and PALS measurements.

To elucidate the molecular basis of the observed charge screening, we performed MD simulations resolving the spatial charge distribution around a single PEI chain with a realistic polymer size (19 kDa). The simulations revealed that the number of condensed ions is significantly higher in CBS than in PBS (Fig. 3e), indicating a weaker association between phosphate ions and PEI. This behavior can also be observed in radial charge density profile (Fig. 3f) where citrate ions assembled into a well-defined shell around the PEI chain, generating a net negative charge density near the polymer interface (Fig. 3e, f). As a result, citrate ions achieved near-complete charge compensation, leading to a cumulative charge approaching zero at long distances, consistent with the stronger charge neutralization observed in PALS measurements (Fig. 3d). In contrast, phosphate ions failed to fully neutralize the positive charges on PEI, reflected in a net positive cumulative charge profile (Fig. 3g) and non-zero zeta-potential in PALS measurements (Fig. 3d).

Further MD simulations in PBS and CBS were performed to elucidate the effect of divalent and trivalent ions on DNA–PEI interactions. A single PEI molecule (degree of polymerization = 28, ~1.2 kDa) was placed in a simulation box containing a 24-base-pair DNA duplex and PEI–DNA binding free energy was quantified in both buffers using umbrella sampling (Fig. 3h). The resulting profiles (Fig. 3i) show that binding is markedly weaker in CBS than in PBS, demonstrating that citrate ions significantly attenuate PEI–DNA affinity.

Strong PEI–DNA interactions in PBS impede structural rearrangement, leading to kinetically trapped, irregular complexes; characteristic of a diffusion-limited aggregation mechanism. In contrast, weaker interactions in CBS allow extensive structural reorganization, facilitating the formation of more spherical complexes. Here, complex growth is governed by the slower association/reorganization or ion replacement step, i.e., reaction-limited aggregation in the citrate-mediated system. Even though PEI carries near-zero net charge in CBS, binding remains spontaneous because displacement of bound citrate ions into bulk solution provides an entropic driving force. Collectively, these results suggest that PBS-mediated assembly proceeds rapidly (short *τ*_*c*_) and is prone to heterogeneity, whereas CBS-mediated assembly is slower (longer *τ*_*c*_) and yields more homogeneous complexes, largely independent of mixing rate (Fig. 3j, k). In further self-assembly simulations (Supplementary Fig. 7), we successfully captured the morphology differences of pDNA/PEI complex depending on charge neutralization by ions, matching the experimental observations (Fig. 2e–g).

### Robustness of the kinetic assembly in CBS under different mixing conditions and scales

**Fig. 4:**
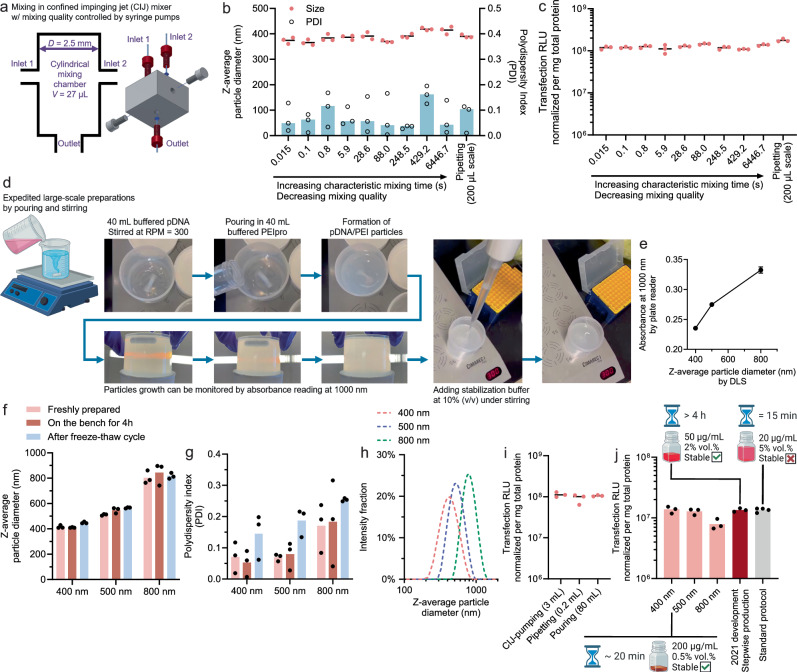
CBS-mediated assembly of stable, concentrated pDNA/PEI particles in different preparation scales. **a** The confined impinging jet (CIJ) mixer (coupled with syringe pumps) was used to vary the flow rate and characteristic mixing time (or mixing quality) of the buffered pDNA solution and the buffered PEIpro solution. **b**, **c** The size and PDI (**b**), and transfection efficiency (**c**) of 400-nm particles loaded with 10% (w/w) luciferase plasmid produced with different mixing quality, as controlled by the CIJ mixer or performed directly by pipetting. **d** The representative images of a particle production process executed at a scale of 80 mL and a pDNA concentration of 200 μg/mL, using easy-to-access labware under magnetic stirring and manual mixing. **e** Size growth monitored by measuring the absorbance of 100 μL of growing particles in a 96-well plate using a plate reader, which completes within seconds. Data are presented as mean ± SD. **f**, **g** The on-bench (4 h) and shelf (after a freeze-thaw cycle) stability of the particles produced at different sizes with an 80-mL scale in terms of the particle size (**f**) or the PDI (**g**) as assessed by DLS. **h** The size distribution of the particles produced on an 80-mL scale. **i** The transfection efficiency of 400-nm particles produced on an 80-mL scale compared to 400-nm particles prepared using the CIJ mixer or by pipetting. **j** The transfection of pDNA/PEIpro particles produced at different sizes with an 80-mL scale compared to 400-nm particles produced by our previously developed stepwise method or compared to particles prepared according to the manufacturer’s protocol for PEIpro. The insets specify the time needed to prepare the particles, the pDNA concentration, the relative culture volume ratio, and stability. Data are presented as mean (*n* = 3 technical replicates from one particle preparation).

Next, we tested the hypothesis that the slower PECn kinetics rendered by citrate ions can ensure uniform assembly under different mixing conditions. To quantitatively vary the mixing quality, we used a confined impinging jet (CIJ) mixer^34^ coupled with digital syringe pumps to control the flow rate during mixing. With a defined geometry (Fig. 4a), we previously demonstrated that the characteristic mixing time *τ*_*m*_ correlates with the total flow rate (Supplementary Table 1)^27,35^. We varied the total flow rate to mix PEIpro solution with pDNA solution diluted by CBS to obtain a characteristic mixing time ranging from 15 milliseconds to minutes, pipetted the stabilization buffer into the mixture at the same post-mixing time, and finally vortexed the mixture to fully stabilize the particles. Using this experimental setup to assemble the 400-nm pDNA/PEIpro particles by CBS, the PDI of the particles remained close to 0.1 regardless of the mixing rate (Fig. 4b). All particles generated showed consistent transfection efficiency using luciferase pDNA as a reporter to transfect HEK293T cells (Fig. 4c).

To fully demonstrate the wider applicability of this assembly method and scalability, we prepared pDNA/PEIpro at a batch size of 80 mL and selected a simple pouring method to mix 40 mL of PEIpro solution with 40 mL of pDNA solution in a 250-mL polypropylene (PP) beaker using a magnetic stirrer operating at 300 rpm (Fig. 4d). This mixing method is far less controlled than a typical pump-operated mixing condition. We also monitored particle size growth by measuring the absorbance at 1000 nm using a plate reader (Fig. 4e). Using a working curve reliably correlating DLS size of the particle suspension, linearly proportional to the absorbance at 1000 nm, we prepared stabilized particles at different target average sizes by pipetting the stabilization buffer into the suspension (Fig. 4d). Using such set-up, we generated three batches of particles with an average size of 400, 500 nm, and 800 nm, respectively. We verified their stability on the bench and after a freeze-thaw cycle by DLS (Fig. 4f). Low PDI values of around 0.1 were confirmed for the 400-nm and 500-nm particles (Fig. 4g). Combined with the unimodal size distribution of these particles (Fig. 4h), these results suggest that a high degree of uniformity was achieved with this crude scale-up mixing setup. The freeze-thaw process seemed to slightly increase PDI to around 0.2 (Fig. 4g). More importantly, the particles produced at this scale showed consistent transfection efficiency using a luciferase reporter pDNA in HEK293T cells, compared with particles generated at smaller scales by either pipetting or highly efficient CIJ mixing (Fig. 4i). At this production scale, our previously developed stepwise particle assembly method^20^ would take over 4 h to complete the production. The preparation protocol described here using the CBS buffer shortened the total assembly time to 15–45 min, which is substantially favorable.

### Transfection efficiency of the CBS-assembled shelf-stable pDNA/PEI particles

To benchmark our method of producing highly concentrated pDNA/PEIpro particles, we compared the transfection efficiency of CBS-assembled particles at 200 μg/mL, >10× above any published PEI method, with those produced using two industry-standard methods: (1) our previously published stepwise protocol, which generates pDNA/PEIpro particles at 50 μg/mL that was tested in LVV production^19^, and (2) the manufacturer’s PEIpro protocol, which generates non-stable complexes at 20 μg/mL. Despite the differences in preparation and concentration, the transfection efficiencies were comparable across all three formulations (Fig. 4j). This demonstrates that our method preserves functional performance, while offering distinct advantages of high operational stability and shelf stability, high pDNA concentration (up to 200 μg/mL), and a shorter preparation time of ~20 min.

**Fig. 5:**
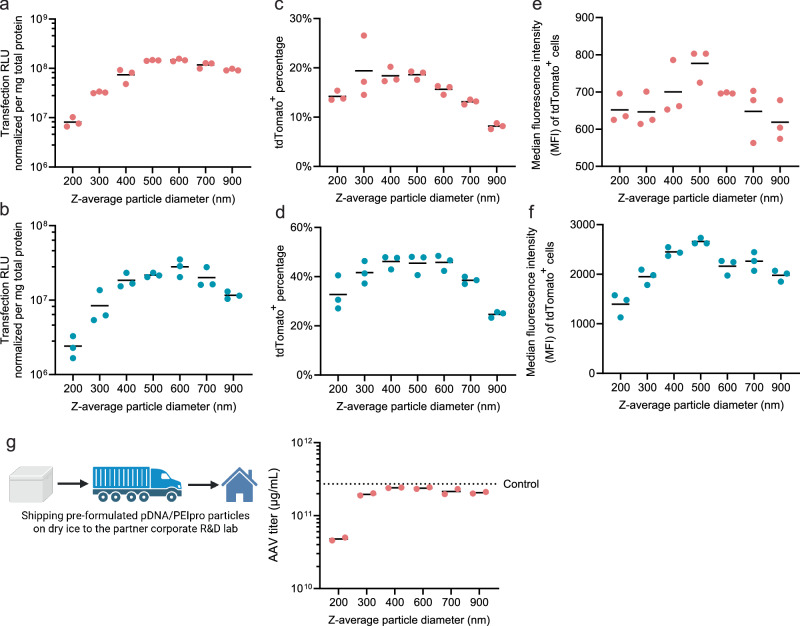
Transfection efficiency and rAAV production using CBS-assembled, concentrated, and stable pDNA/PEI particles. **a**, **b** Transfection efficiency by different-sized particles loaded with 10% (w/w) luciferase plasmid, assessed at 24 h after transfection of HEK293T cells (**a**), or at 48 h after transfection of HEK293F cells (**b**). **c**, **d** Transfection efficiency by different-sized particles loaded with 100% tdTomato plasmid in terms of transfection rate (tdTomato^+^), assessed at 24 h after transfection of HEK293T cells (**c**), or at 48 h after transfection of HEK293F cells (**d**). **e**, **f** Transfection efficiency of different-sized particles loaded with 100% tdTomato plasmid in terms of expression level per cell, i.e., median fluorescent intensity or MFI of tdTomato^+^ cells, assessed at 24 h after transfection of HEK293T cells (**e**), or at 48 h after transfection of HEK293F cells (**f**). **g** rAAV titer from bioreactor production at Biogen Inc., transfected with pre-formulated stable pDNA/PEIpro® particles at different sizes that were shipped on dry ice, thawed at ambient temperature for 2 h, and tested. Data are presented as mean (*n* = 3 technical replicates from one particle preparation).

We then evaluated the size-dependent transfection efficiency of CBS-assembled particles in HEK293T (adherent culture) and HEK293F (suspension culture) cells using luciferase and tdTomato reporter plasmids. The transfection efficiency measured by the total luciferase expression level (Fig. 5a, b), percentage of tdTomato^+^ cells characterized by flow cytometry (Fig. 5c, d), and average gene expression level per cell by the median fluorescent intensity (MFI) of tdTomato^+^ cells (Fig. 5e, f) revealed that the peak transfection efficiency was mediated by pDNA/PEI particles with an average size of 400 to 500 nm, showing the size-dependent profile consistent with our prior findings^21^. Importantly, cell viability assays showed no significant cytotoxicity across particle sizes compared with the negative control, at the dose used for transfection studies (Supplementary Fig. 8), indicating that the observed differences in transfection performance are not confounded by cytotoxicity effects. Additionally, particles incubated in cell culture medium with or without 10% FBS at 37 °C did not result in catastrophic aggregation over the relevant time window (Supplementary Fig. 9).

Furthermore, to evaluate multi-plasmid delivery capability, GFP- and tdTomato-encoding plasmids were co-formulated at defined input ratios using the 400-nm CBS-assembled nanoparticles. Single-cell fluorescence analysis by flow cytometry revealed a composition-dependent shift in the proportions of GFP⁺ and tdTomato⁺ cells as the input plasmid ratio was varied (Supplementary Fig. 10), showing a near-linear trend without preferential enrichment of either plasmid. These results indicate that the citrate-mediated kinetic assembly strategy does not introduce selective encapsulation or delivery bias in multi-plasmid systems.

### Third-party validation: AAV production using CBS-assembled shelf-stable transfection particles

To assess translational relevance, we collaborated with Biogen Inc. to test our method in an industrial AAV production setting. We used a plasmid cocktail developed by Biogen Inc. (USA, NASDAQ: BIIB) and formulated pDNA/PEIpro particles with an average size ranging from 200 to 900 nm in our Johns Hopkins University laboratory in Baltimore MD, using the CBS assembly protocol. The assembled particles were stored at –80 °C before shipment on dry ice to the Biogen laboratory in Cambridge, MA, where the particles were stored at –80 °C until use. On the day of experiments, the particles were thawed at ambient temperature for 2 h and then added to a benchtop bioreactor according to Biogen’s standard AAV production protocol. These particles with an average size of 300 to 900 nm yielded a similar AAV titer that matched the control level (Fig. 5g), which represents a typical titer obtained from the optimized Biogen protocol. In addition to viral genome titers, genome-to-capsid titer ratios were quantified as an indicator of coordinated intracellular delivery and expression of packaging plasmids. Across multiple formulation concentrations, citrate-mediated PEI–pDNA complexes yielded genome-to-capsid ratios comparable to or exceeding those of the industry control, supporting balanced multi-plasmid delivery during AAV assembly (Supplementary Fig. 11). These results demonstrated the functionality, shelf stability, and bench stability of the pDNA/PEIpro particles assembled by CBS.

## Discussion

Our findings establish kinetic gating by multivalent counterions as a general principle for assembling polyelectrolyte nanocomplexes under manufacturing-relevant conditions. Where earlier citrate studies provided useful but short-lived stability at dilute DNA loadings, we show that modulating the early-stage complexation rate unlocks ten-fold higher plasmid concentration, high room-temperature stability, and full stability after cryo-storage. Because kinetic gating relies only on buffer composition, it is immediately transferable to other polyelectrolyte therapeutics (such as siRNA-polyplexes and cell-penetrating peptide systems), positioning the concept well beyond PEI–pDNA chemistry.

Using the citrate-buffered saline developed, we consistently produced 400–600 nm pDNA/PEI particles within 20–40 min at 200 μg/mL, 10–20 times the typical concentration used in industry. This allowed a substantial reduction in the bioreactor dosing volume of the transfection particles. The CBS-assembled particles also exhibit a high degree of on-bench stability for at least 4 h at ambient temperature, good shelf stability under cold storage at –80 °C, and freeze-thaw stability. More importantly, this CBS-mediated assembly method is robust across different mixing methods and batch scales. This platform may simplify liquid-handling logistics, reduce process variability, and enhance manufacturing flexibility, making it suitable for broad adoption in viral vector production across various batch-size scales. These features are particularly favorable for performing transfection in large-scale AAV production to develop more robust processes and to potentially reduce the cost of AAV manufacturing. Beyond AAV production, this nanoparticle assembly protocol may have broad utility in enhancing the process robustness and efficiency of viral vector production at industry scales.

Together, these advances convert multivalent ions such as citrate into an engineered kinetic modulator, offering both a practical solution to current manufacturing bottlenecks and a framework for future ion-mediated assembly strategies.

## Methods

### Preparation of shelf-stable and high-concentration pDNA/PEI particles

Plasmid DNA (gWiz-Luc, 6732 bp or tdTomato plasmid; both from Aldevron) was dissolved at 440 μg/mL in a pDNA dilution buffer containing 19% (w/w) trehalose. Poly(ethyleneimine) (PEIpro, Polyplus Sartorius, France) was similarly dissolved at 580.8 μg/mL in a PEI dilution buffer with 19% (w/w) trehalose. For kinetic control studies shown in Figs. 2–4, the dilution buffers included either phosphate-buffered saline (PBS; 137 mM NaCl, 2.7 mM KCl, 10 mM Na₂HPO₄, 1.8 mM KH₂PO₄) or citrate-buffered saline (CBS; 140 mM NaCl, 4 mM trisodium citrate, pH 7.0). For rAAV production, the buffers were optimized for pDNA cocktails from Biogen. The pDNA and PEI solutions were mixed at a 1:1 volume ratio, and the concentration of PEI was adjusted to achieve a nitrogen-to-phosphate (N/P) ratio of 5.5. When particles reached the target size monitored by optical density, a stabilization buffer containing trehalose and an acidic pH was added at a 10% v/v ratio to stop further size growth. Final particle suspensions contained 200 μg/mL pDNA and were either used immediately or stored at –80 °C.

### Control of mixing quality using a confined impinging jet (CIJ) mixer

To study the effect of mixing kinetics on particle assembly, we used a CIJ mixer that we previously developed^22,29^. The PEI and pDNA solutions were loaded into separate syringes and injected into the CIJ device at controlled flow rates using a digital syringe pump (NE-4000, New Era Pump Systems). Flow rate determined the characteristic mixing time (*τ*_*m*_), allowing systematic evaluation of mixing effects on particle size and uniformity.

### Benchtop-scale particle production

For scale-up validation experiments described in Fig. 4d–j, 40 mL of pDNA solution was combined with 40 mL of PEI solution in a 250-mL polypropylene beaker under constant stirring at 300 rpm using a standard lab stirrer. Particle growth was monitored by measuring absorbance at 1000 nm using a 96-well plate reader. A calibration curve was established to correlate absorbance with DLS-derived particle size. Once particles reached the desired size (e.g., 400, 500, or 800 nm), stabilization buffer was added as described above.

### Characterization of the assembled pDNA/PEI particles

During complexation or after stabilization, the size of the particles was characterized by dynamic light scattering (DLS) using a Zetasizer ZS90 (Malvern, USA) at a 90° scattering angle. Z-average diameters (*D*_*Z*_) were reported throughout the study. The polydispersity index (PDI) was directly given by the instrument and is defined as ${PDI}={\left(\triangle /{D}_{z}\right)}^{2}$, in which Δ is the standard deviation of the size distribution. For morphology analysis, particles were deposited on glow-discharged lacey carbon TEM grids, negatively stained with 2% uranyl acetate, air-dried for 24 h, and imaged using a Talos F200C transmission electron microscope (Thermo Fisher Scientific).

### Cell culture and transfection experiments

For monolayer culture studies, HEK293T cells (CRL-3216™, American Type Culture Collection, ATCC, USA, maintained in DMEM supplemented by 10% FBS and 2 mM L-glutamine, at 37 °C, 5% CO_2_, and saturated humidity) were seeded at a cell density of 100,000 cells/well in a 24-well plate, 1 day prior to transfection. Transfection particles at a DNA concentration of 200 μg/mL containing 10% (w/w) luciferase plasmid with 90% (w/w) non-coding plasmid or containing 100% tdTomato plasmid were diluted in Opti-MEM medium. The medium in each well of the plate was then replaced by particle-containing Opti-MEM. The stabilized particles were dosed into the cells at 1 μg DNA per well, and incubated with the cells for 4 h, followed by medium exchange with fresh cell culture medium and a subsequent culture of 44 h. Cells were then lysed with 200 μL reporter lysis buffer (Cat. #E4030, Promega Co.), and the lysates underwent a freeze-thaw cycle (–80 °C to ambient temperature). For luciferase measurements, 20 μL of each sample was mixed with 100 μL luciferase substrate (Cat. #E1483, Promega Co.) and the luminescence signal was measured by a luminometer. For tdTomato fluorescence assessment, the cells were detached by Trypsin-EDTA and stained with LIVE/DEAD™ Fixable Aqua Dead Cell Stain Kit (Thermo Fisher Scientific Inc.) for viability assessment before being analyzed by an Attune™ NxT Flow Cytometer (Thermo Fisher Scientific Inc.). The representative flow cytometry gating strategy is shown in Supplementary Fig. 12.

For suspension culture studies, HEK293F cells (R79007, Thermo Fisher Scientific, USA; maintained in FreeStyle 293 medium, at 37 °C, 8% CO_2_, and saturated humidity) were seeded at 1,000,000 cells/well in a 12-well plate, on the day of transfection. Stabilized particles at a DNA concentration of 200 μg/mL containing 10% luciferase plasmid with 90% non-coding plasmid, or 100% tdTomato plasmid, were added directly into the cell suspension to reach a dosage of 1 μg DNA per well. Cells were incubated with the particles for 48 h until analysis. For luciferase assessment, cells were collected from the plates, pelleted by centrifugation at 300 × *g* for 5 min, and then lysed with 500 μL reporter lysis buffer. The luminescence assay was carried out using the same protocol as described above; the tdTomato fluorescence assessment using flow cytometry was conducted using the same protocol as described above. Representative flow cytometry gating strategies are provided in Supplementary Fig. 12.

### Statistical analysis

For in vitro transfection experiments, the sample size was *n* = 3 unless otherwise specified. For rAAV titer tests provided by Biogen Inc., *n* = 2. Nanoparticle size and zeta potential measurements by DLS were obtained from repeated measurements of each sample. Distinct biological samples were collected as replicates for all in vitro experiments. Data are presented as mean unless stated otherwise. Statistical significance was determined using two-way ANOVA with Šídák’s multiple comparisons. Exact P values are reported in the figure legends where applicable.

### Molecular dynamics (MD) simulations

MD simulations presented in this manuscript were performed in GROMACS^36^ with Martini force field parameters^33,37,38^. These coarse-grained simulations allowed us to run for longer and relatively large systems, which would not have been possible with atomistic simulations. We used Salassi et al.^37^ parameters for modeling citrate ion and Mahajan and Tang^38^ parameters for PEI where 40% of the monomers were charged. Fifty small PEIs (14-monomers) were randomly placed in a 20 × 20 × 20 nm^3^ simulation box with 140 mM NaCl and added 10 mM of phosphate or citrate ions. First, energy minimization was performed on the systems, followed by initial equilibration under NVT ensemble for 50 ns with 10 fs timesteps. The final equilibration simulation was performed for 5 μs with 20 fs timestep. Neighbor searching was performed up to a cut-off distance of 1.2 nm using the Verlet particle-based approach. The potential-shift method was applied for the short-range Lennard-Jones (LJ) 12-6 interactions at a cut-off of 1.2 nm. The reaction-field method was used for calculations of the non-bonded interactions between charge beads (cutoff 1.2 nm). The dielectric constant was fixed at 15 according to the Martini model. The bonds were constrained with the LINCS algorithm^39^. The temperature of the system was maintained using the v-rescale algorithm at a reference temperature of 298 K. The isotropic Parrinello-Rahman barostat was utilized with the reference pressure of 1 bar^40^. Ovito was used for clustering and visualizations^41^, and a cluster cutoff of 0.72 nm was selected based on the first valley of the Martini bead radial distribution function^33^. To measure the aggregation process, we calculated the number-average cluster size (CSN) as ${{{\rm{CSN}}}}=\sum N{{{\rm{\cdot }}}}M/\sum M$, where N is the cluster size, and M is the concentration of the clusters. If at least two monomers were within 0.72 nm of each other, we defined them as forming a cluster and then normalized CSN with the total number of PEI added to the simulation box. Similar simulation procedures were followed for simulating large PEI, although only 1 PEI was placed in the simulation box.

For calculating the free energy of pDNA/PEI binding, we first placed a 24 bp DNA (Sequence: dCdGdCdGdAdAdTdTdCdGdCdGdCdGdCdGdAdAdTdTdCdGdCdG) in a simulation box and added a PEI near it. After 1 μs of equilibration, we pulled the PEI from the DNA in the radial direction using a constant force. From different distances between DNA and PEI, we selected reaction coordinates that were 1.5 Å apart. We performed umbrella sampling in those coordinates using a force constant of 1000 kJ/mol/nm^2^ and used the WHAM algorithm to get the binding free energy^42^. Each reaction coordinate was simulated for 700 ns, of which the last 500 ns of data were used during the WHAM algorithm.

The methods to perform the MD simulations shown in Supplementary Fig. S3 are described in **Supplementary Methods**.

### Reporting summary

Further information on research design is available in the Nature Portfolio Reporting Summary linked to this article.

## Supplementary information

Supplementary_Information

Reporting Summary

Transparent Peer Review file

## Source data

Source Data

## Data Availability

Source data underlying Figs. 2–5 are available in Zenodo under accession code 10.5281/zenodo.19636012. Figure 1 is a schematic illustration and has no associated source data. All other data supporting the findings of this study are available within the paper and its Supplementary Information. Details of the MD simulation methods are provided in the Supplementary Methods. Source data are provided with this paper.
