# Supplementary material for: Trivalent ions kinetic-gating for producing high-concentration and shelf-stable plasmid DNA/PEI particles
Source: Nat Commun. 2026 Jun 2;17:7107. doi: 10.1038/s41467-026-73921-4 (PMC13392119; doi:10.1038/s41467-026-73921-4)
Supplement: Supplementary file 1 — Supplementary_Information [file 41467_2026_73921_MOESM1_ESM.pdf]

Supplementary Information for

Trivalent Ions Kinetic-Gating for Producing High-Concentration and Shelf-Stable Plasmid DNA/PEI Particles

Jinghan Lin[1,2,3,†], Yizong Hu[1,2,4,9,†,*], Turash H. Pial[2], Kailei D. Goodier[1,2,3], Di Yu[1,2,4], Paetra Brailsford[5], Maria Choi-Ali[5], Jonathan T. Feng[1,3,4], Sixuan Li[1,6], Yining Zhu[1,3,4], Jingyao Ma[1,2,3], Leonardo Cheng[1,3,4], Xiaoya Lu[1,2,3], Nicole Korinetz[7], Marine Guise[8], Tza-Huei Jeff Wang[1,4,6], Tine Curk[1,2], Hai-Quan Mao[1,2,3,4,*]

[1]Institute for NanoBioTechnology, Johns Hopkins University, Baltimore, MD, USA.

[2]Department of Materials Science and Engineering, Johns Hopkins University, Baltimore, MD, USA.

[3]Translational Tissue Engineering Center, Johns Hopkins University School of Medicine, Baltimore, MD, USA.

[4]Department of Biomedical Engineering, Johns Hopkins University School of Medicine, Baltimore, MD, USA.

[5]Biogen Inc., Cambridge, MA, USA.

[6]Department of Mechanical Engineering, Johns Hopkins University, Baltimore, MD, USA.

[7]Department of Chemical and Biomolecular Engineering, Johns Hopkins University, Baltimore, MD, USA.

[8]Polyplus Sartorius, Illkirch-Graffenstaden, France.

[9]Present address: David H. Koch Institute for Integrative Cancer Research, Massachusetts Institute of Technology, Cambridge, MA, USA.

[†]These authors contributed equally to this work.

*Corresponding authors: Yizong Hu (yizonghu@mit.edu) and Hai-Quan Mao (hmao@jhu.edu).

Supplementary Methods

MD simulations presented in this Supplementary Information are performed in the LAMMPS molecular dynamics package with reduced Lennard-Jones (LJ) units. The system consists of PEI, DNA, and ions (phosphate and citrate), modeled using a coarse-grained bead-spring representation. Non-bonded interactions are modeled using the Weeks–Chandler–Andersen (WCA) potential:

$$V(r, b) = \begin{cases} 4\epsilon_{LJ}[(b/r)^{12} - (b/r)^6 + 1/4], & r \leq r_c \\ 0, & r > r_c \end{cases}$$

We used $\sigma = 0.32$ nm as the unit of length and $\epsilon_{LJ} = 1$ sets the energy scale. $b_{DNA,DNA} = 3.12\sigma$, $b_{DNA,PEI} = 3.12\sigma$ and all other bead pairs: $b = \sigma$. The cutoff for all interactions is fixed at $r_c = 2^{1/6}b$, ensuring purely repulsive interactions. To capture the intrinsic rigidity of DNA and PEI, angular potential was applied according to respective persistence length. Electrostatic interactions were computed using the Debye–Hückel approximation, with a screening length corresponding to an ionic strength of ~100 mM. Additional multivalent ions (10 mM concentration) were modeled explicitly: divalent ions as single charged beads, and citrate ions as linear trimers of three charged beads connected by stiff harmonic bonds, capturing their trivalent, multidentate nature. Simulation time step was 0.002. All simulations used the velocity-Verlet integrator with a Langevin thermostat. Periodic boundary conditions were applied in all directions.

For supplementary Fig. 7a. The system contained a 300 bp DNA chain and PEI chains with 80 monomers each. Initial equilibration was performed in the absence of attractive DNA–PEI interactions to allow PEI complexation with ions (5,000,000 steps). This mimics pre-complexation in solution. Attractive DNA–PEI interactions were then introduced to study complex formation and morphology (10,000,000 steps).

For Supplementary Fig. 7b: Citrate ions were modeled as linear trimers of three charged beads connected by stiff harmonic bonds. The PEI chains contained 80 monomers; Divalent ions were modeled as single charged beads.

Supplementary Figures

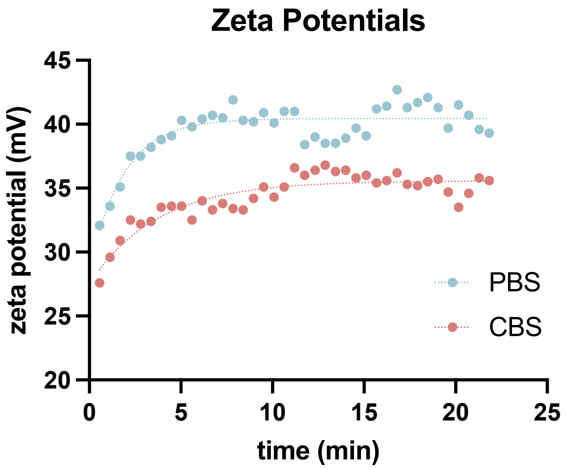

Supplementary Fig. 1. Evolution of zeta potential during PEI–pDNA particle assembly in PBS and CBS.

Time-resolved zeta potential measurements of particles assembled in PBS and CBS, monitored immediately after mixing.

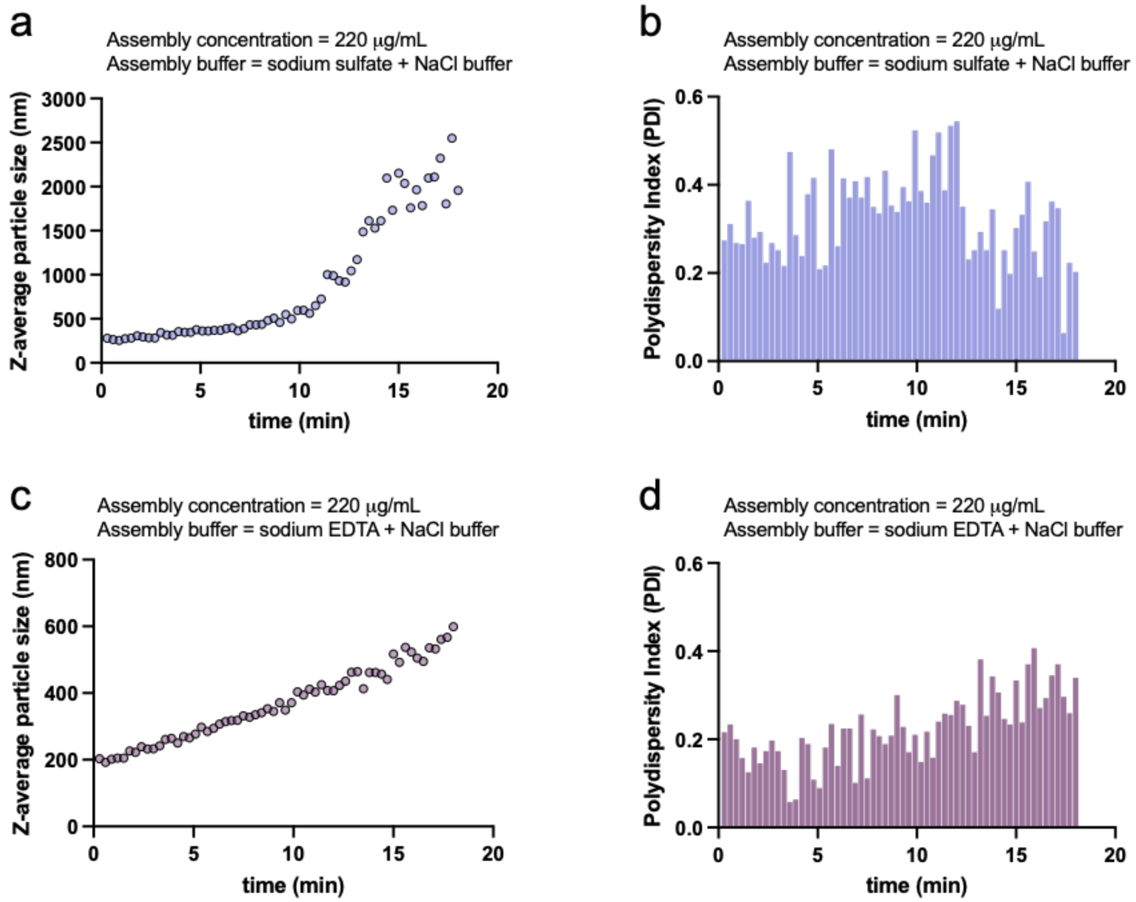

Supplementary Fig. 2. Effect of alternative multivalent anions on PEI–pDNA assembly kinetics.

(a) Size evolution of PEI–pDNA particles assembled in Na_2SO_4-containing buffer (pH 7.0). **(b)** Corresponding polydispersity index (PDI) over time in Na_2SO_4 buffer. **(c)** Size evolution of PEI–pDNA particles assembled in sodium EDTA-containing buffer (pH 7.0). **(d)** Corresponding PDI over time in EDTA buffer. All measurements were performed by DLS immediately after mixing under identical formulation conditions.

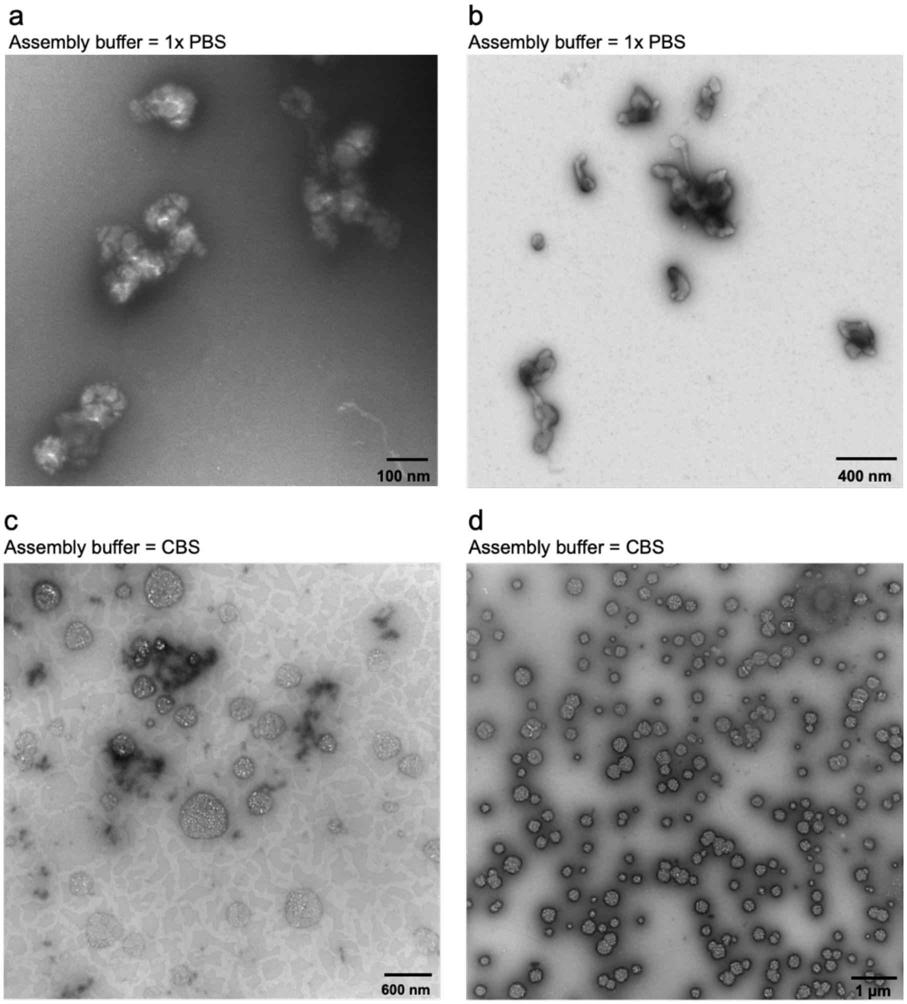

Supplementary Fig. 3. Transmission electron microscopy (TEM) characterization of PEI–pDNA particles assembled in PBS and CBS.

Representative TEM images of particles assembled in PBS **(a, b)** and CBS (**c, d)** at two different magnifications. PBS-assembled particles exhibit more irregular and heterogeneous structures, whereas CBS-assembled particles display more spherical and uniform morphology. Scale bars are indicated in each panel.

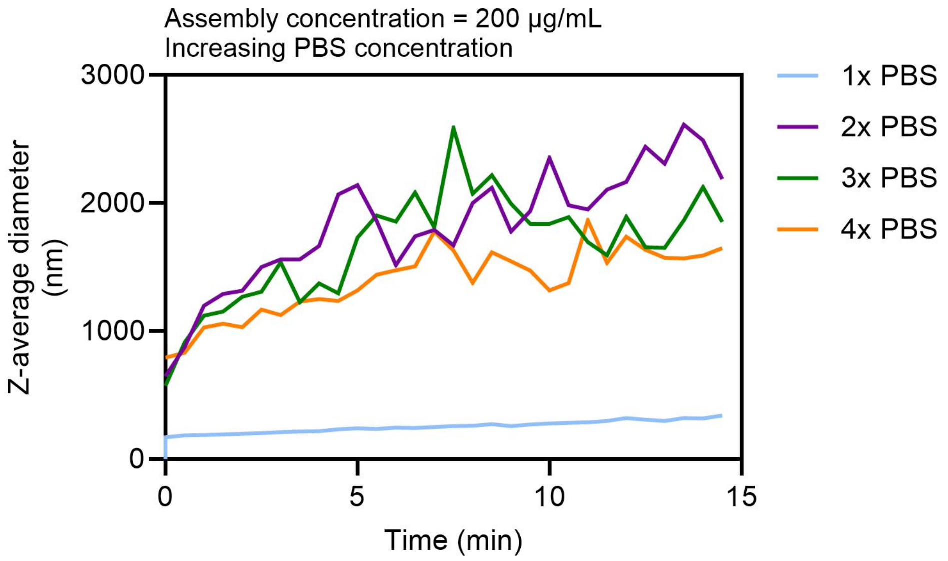

Supplementary Fig. 4. Particle growth at a pDNA concentration of 200 µg/mL mediated by different concentrations of PBS as a buffer.

While increasing the concentration of PBS enhanced charge screening by the increased ionic strength from monovalent and divalent ions, it did not achieve the steady, uniform particle assembly as mediated by citrate-buffered saline.

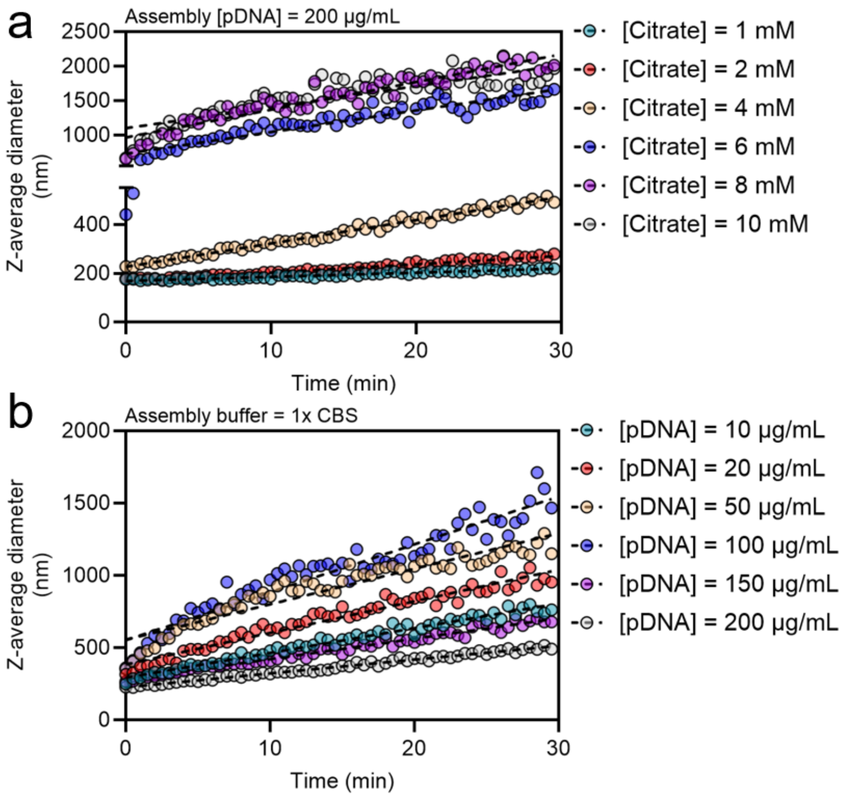

Supplementary Fig. 5. NPa kinetics

The effects of **(a)** plasmid DNA concentration [pDNA] and **(b)** citrate concentration [Citrate] added to the phosphate buffer on the nanoparticle assembly (NPa) process following polyelectrolyte complexation (PECn) step.

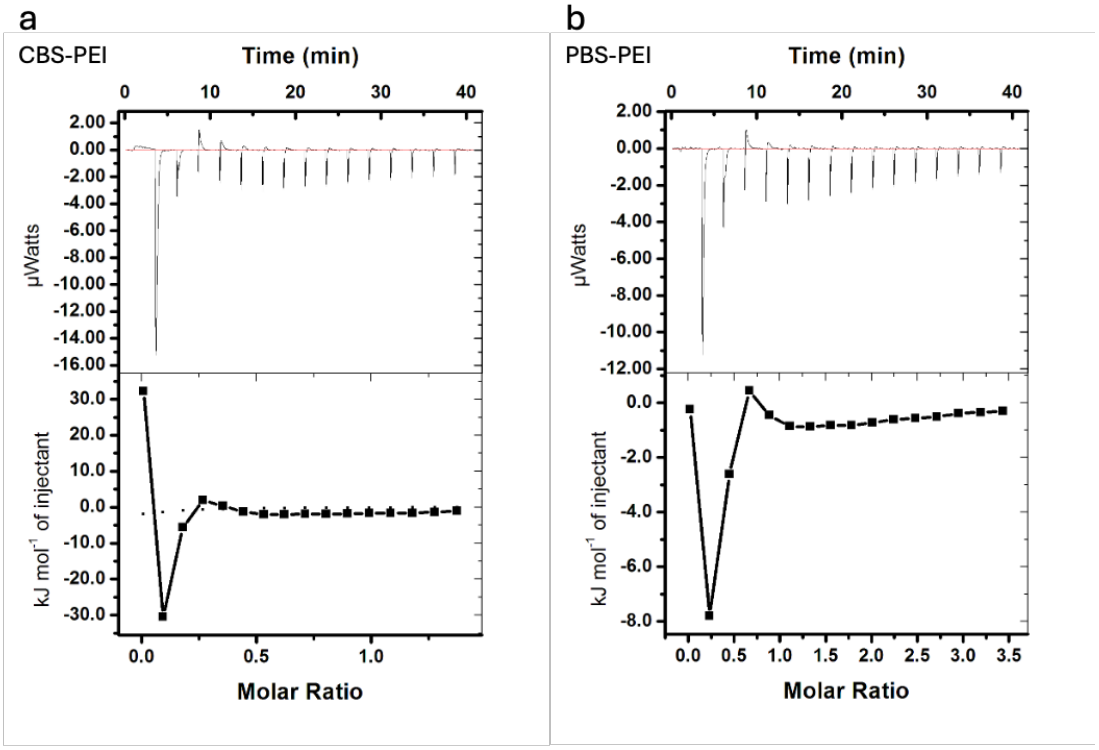

Supplementary Fig. 6. Isothermal titration calorimetry (ITC) analysis of buffer–PEI interactions.

Representative ITC thermograms (upper panels) and corresponding integrated heat profiles (lower panels) for titration of **(a)** CBS and **(b)** PBS into PEI solution. Raw injection heat signals are shown as differential power (μW) versus time, and integrated heats are plotted as a function of molar ratio of buffer ions to PEI. Citrate-containing buffer produces substantially larger heat changes compared to PBS under matched conditions, indicating stronger and more cooperative interactions between citrate ions and protonated PEI. Integrated heat profiles reflect the multivalent, non-stoichiometric nature of PEI–ion association.

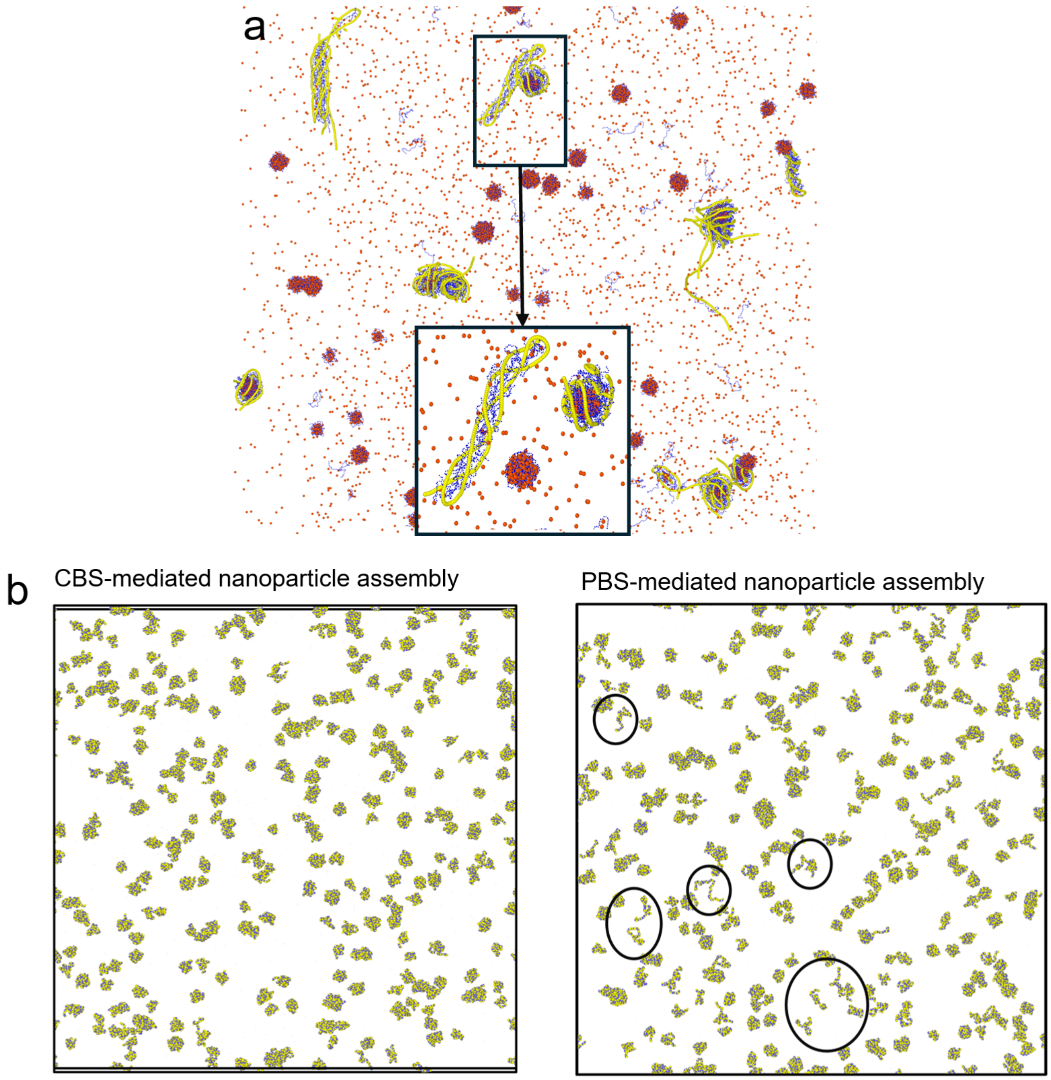

Supplementary Fig. 7. Molecular dynamics simulations for nanoparticle assembly (NPa) step following the initial pDNA-PEI polyelectrolyte complexation (PECn) step.

(a) PEI (blue), pDNA (yellow), and anions (red) were included as the three main components in the system. Over time, we observed the aggregation between anions and PEI molecules, and then some portions of anions were replaced by DNA molecules, which provide stronger binding with PEI due to a higher density of negative charges. **(b)** The incorporation of the trivalent anions of citrate in the PEC resulted in the formation of assembled particles with a more regular, spherical shape, which further accelerates the aggregation between PEC seeds as expected from the DLVO theory, eventually leading to submicron particles. In contrast, PBS-mediated assembly resulted in the formation of assembled particles with more irregular shapes. These simulation findings are consistent with TEM observations.

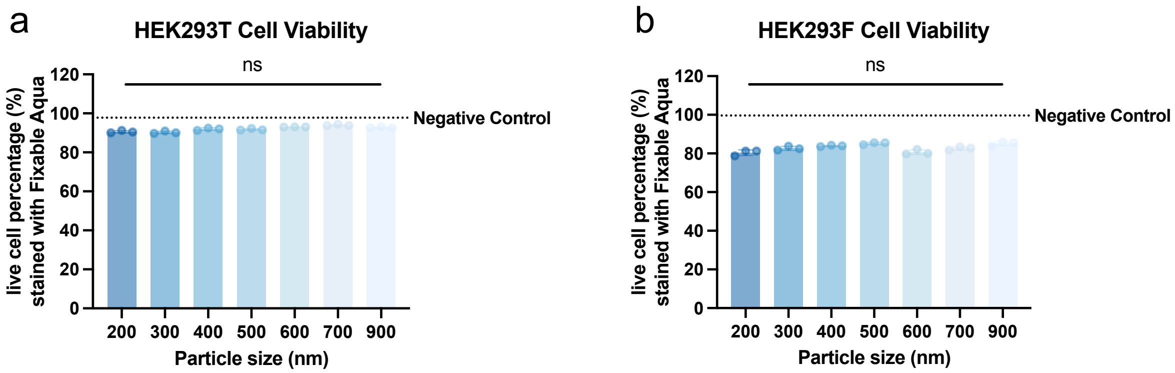

Supplementary Fig. 8. In vitro cytotoxicity assessment of PEI-pDNA particles.

Cell viability was evaluated following treatment with indicated PEI-pDNA particles under the same dosing conditions used for transfection experiments on **(a)** HEK293T cells, **(b)** HEK293F cells. Viability was quantified using LIVE/DEAD Fixable Aqua staining and negative control is shown as dotted line. Statistical analysis was performed using one-way ANOVA followed by Dunn's multiple comparisons test. No statistically significant reduction in cell viability was observed across groups. Data are presented as mean ± s.d. (n = 3).

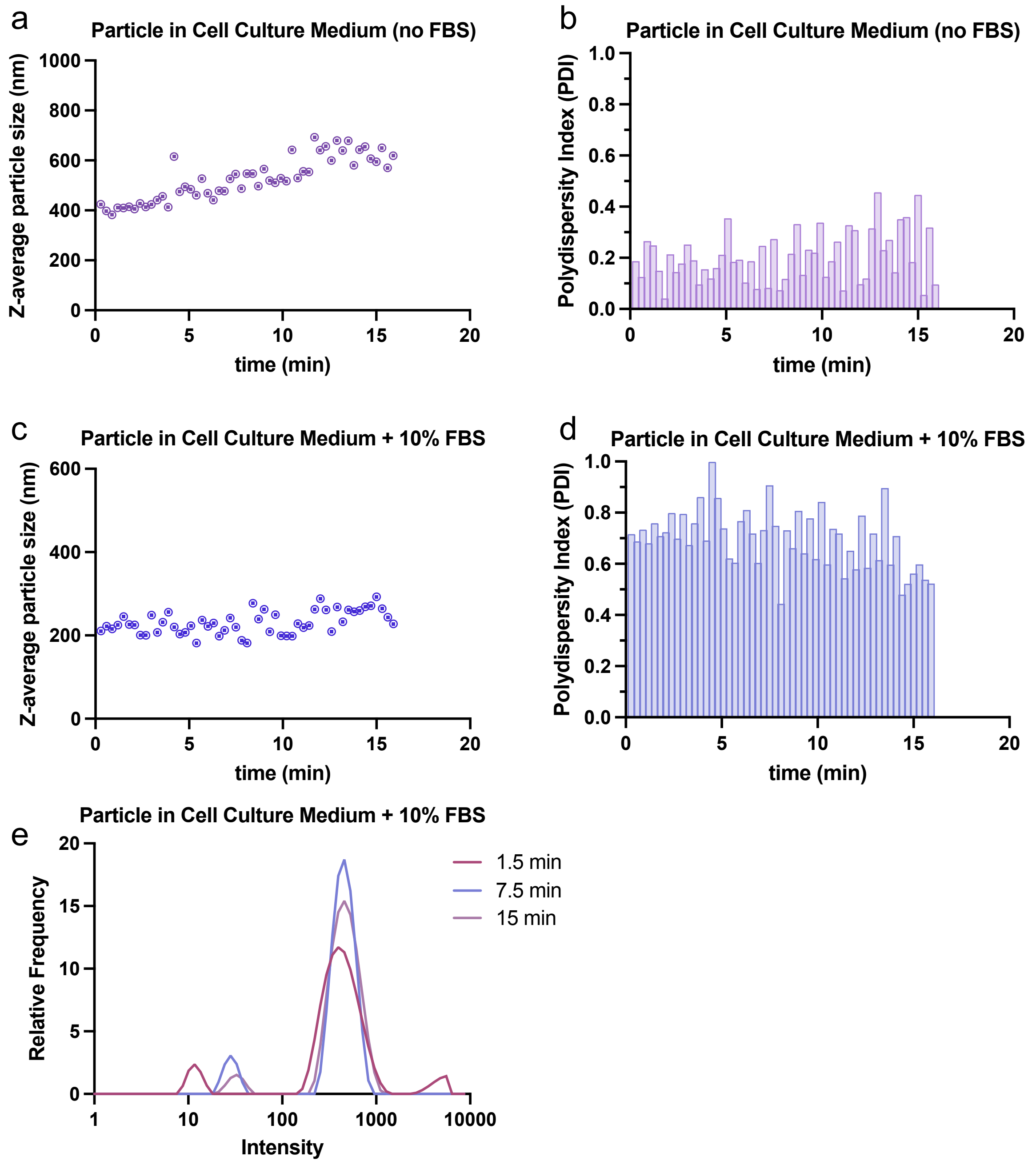

Supplementary Fig. 9. Size and polydispersity index (PDI) of ~400 nm PEI–pDNA particles incubated in cell culture medium at 37 °C, with and without 10% fetal bovine serum (FBS), measured by dynamic light scattering (DLS).

Z-average hydrodynamic diameter of particles incubated in cell culture medium **(a)** without FBS and **(c)** with 10% FBS. Corresponding PDI values are shown for particles incubated **(b)** without FBS and **(d)** with 10% FBS. **(e)** Representative intensity-weighted size distribution of particles incubated in medium containing 10% FBS at early- (1.5 min), mid- (7.5 min) and late- (15 min) time point.

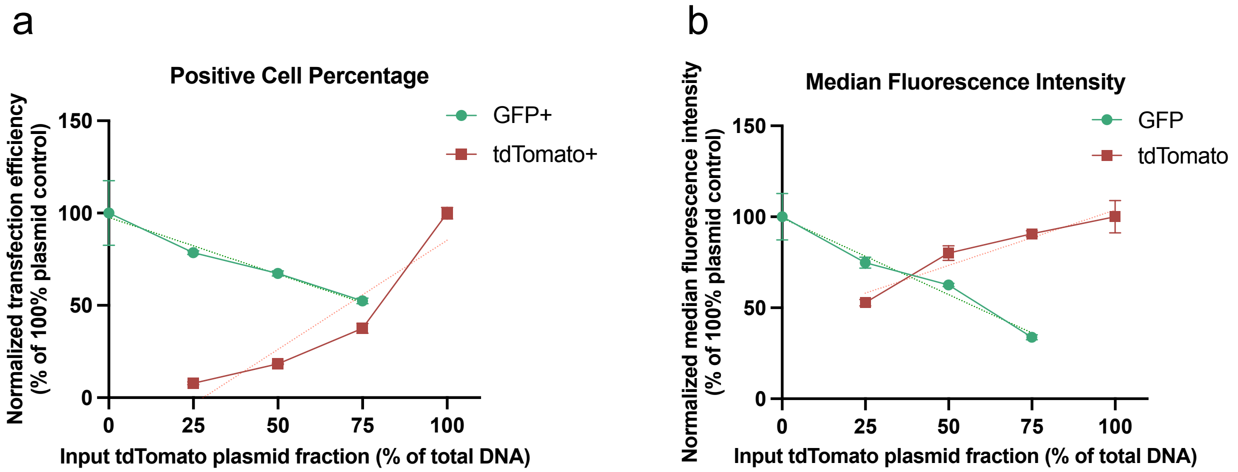

Supplementary Fig. 10. Multi-plasmid co-delivery performance of citrate-mediated PEI–pDNA nanoparticles.

(a) Percentage of GFP^+ and $tdTomato^+$ cells following co-transfection with defined input ratios of GFP- and tdTomato-encoding plasmids formulated using 400-nm CBS-mediated nanoparticles. **(b)** Mean fluorescence intensity (MFI) of GFP and tdTomato signals under the same plasmid mixing conditions. Data are presented as mean ± s.d. (n = 3).

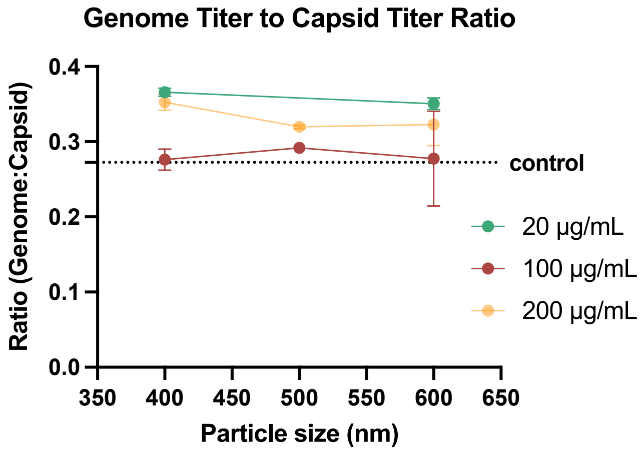

Supplementary Fig. 11. Genome-to-capsid ratio analysis of AAV produced using citrate-mediated PEI–pDNA complexes.

Genome titer and capsid titer were quantified to determine the genome-to-capsid ratio as an indicator of coordinated multi-plasmid delivery during AAV assembly. Data are presented as mean ± s.d. (n = 2).

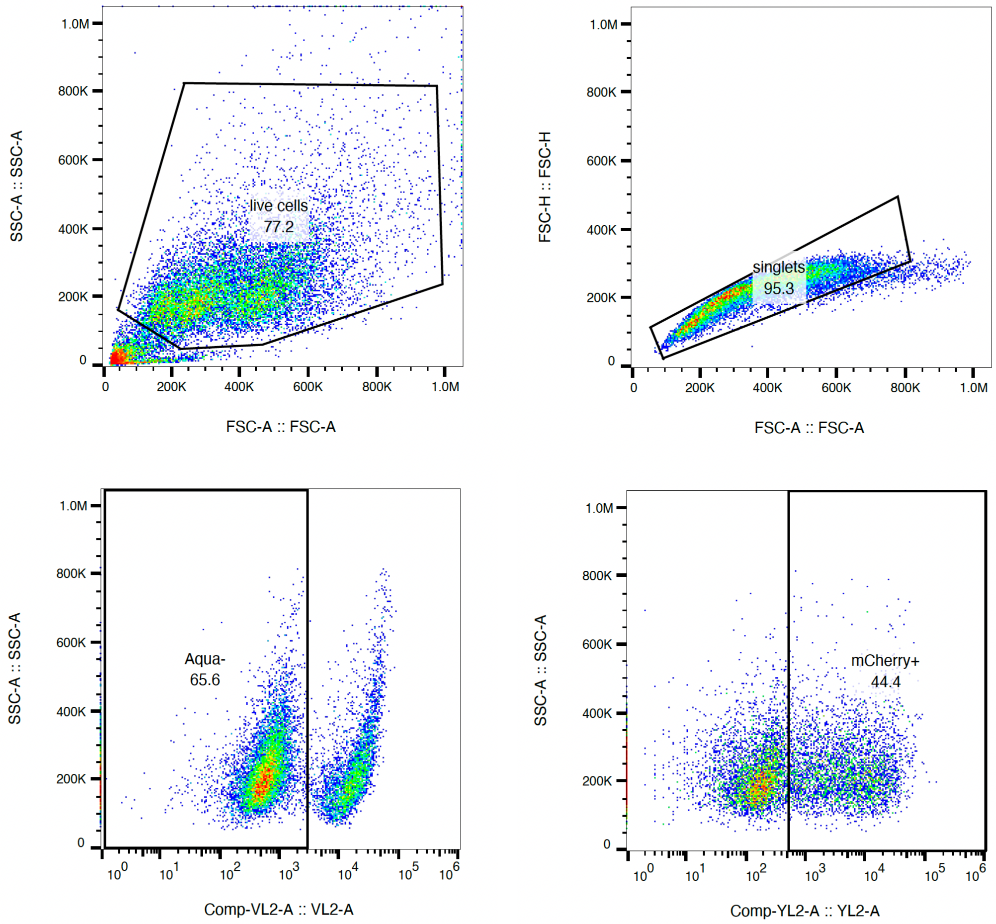

Supplementary Fig. 12. Representative flow cytometry gating strategy used for analysis of cell transfection.

Representative plots showing the sequential gating strategy used to identify the analyzed cell populations. Debris were excluded based on forward- and side-scatter properties, followed by singlets gating where indicated. Final populations were defined according to the fluorescence signals of the corresponding markers, including Live/Dead staining and mCherry expression. Identical gating criteria were applied to all samples within each experiment.

Supplementary Table 1. Theoretical characteristic mixing time correlated with mixing rates.

**Total flow rate in mixing (mL/min)**	**40**	**20**	**10**	**6**	**4**	**3**	**2.3**	**2**	**1**
**Theoretical characteristic mixing time (s)**	0.015	0.1	0.8	5.9	28.6	88.0	248.5	429.2	6446.7
