# Supplementary material for: Trivalent ions kinetic-gating for producing high-concentration and shelf-stable plasmid DNA/PEI particles
Source: Nat Commun. 2026 Jun 2;17:7107. doi: 10.1038/s41467-026-73921-4 (PMC13392119; doi:10.1038/s41467-026-73921-4)
Supplement: Supplementary file 2 — Reporting Summary [file 41467_2026_73921_MOESM2_ESM.pdf]

nature portfolio

Corresponding author(s): Hai-Quan Mao

Last updated by author(s): 2026/04/17

Reporting Summary

Nature Portfolio wishes to improve the reproducibility of the work that we publish. This form provides structure for consistency and transparency in reporting. For further information on Nature Portfolio policies, see our Editorial Policies and the Editorial Policy Checklist.

Statistics

For all statistical analyses, confirm that the following items are present in the figure legend, table legend, main text, or Methods section.

n/a	Confirmed	
☐	☒	The exact sample size (*n*) for each experimental group/condition, given as a discrete number and unit of measurement
☐	☒	A statement on whether measurements were taken from distinct samples or whether the same sample was measured repeatedly
☐	☒	The statistical test(s) used AND whether they are one- or two-sided<br>*Only common tests should be described solely by name; describe more complex techniques in the Methods section.*
☒	☐	A description of all covariates tested
☒	☐	A description of any assumptions or corrections, such as tests of normality and adjustment for multiple comparisons
☐	☒	A full description of the statistical parameters including central tendency (e.g. means) or other basic estimates (e.g. regression coefficient) AND variation (e.g. standard deviation) or associated estimates of uncertainty (e.g. confidence intervals)
☐	☒	For null hypothesis testing, the test statistic (e.g. *F*, *t*, *r*) with confidence intervals, effect sizes, degrees of freedom and *P* value noted<br>*Give P values as exact values whenever suitable.*
☒	☐	For Bayesian analysis, information on the choice of priors and Markov chain Monte Carlo settings
☒	☐	For hierarchical and complex designs, identification of the appropriate level for tests and full reporting of outcomes
☒	☐	Estimates of effect sizes (e.g. Cohen's *d*, Pearson's *r*), indicating how they were calculated

Our web collection on statistics for biologists contains articles on many of the points above.

Software and code

Policy information about availability of computer code

Data collection	GROMACS v.2024 and LAMMPS v.2024 were used to perform molecular dynamics simulation.
Data analysis	Graphpad Prism v10, FlowJo v10 were used to analyze and plot the data.

For manuscripts utilizing custom algorithms or software that are central to the research but not yet described in published literature, software must be made available to editors and reviewers. We strongly encourage code deposition in a community repository (e.g. GitHub). See the Nature Portfolio guidelines for submitting code & software for further information.

Data

Policy information about availability of data

All manuscripts must include a data availability statement. This statement should provide the following information, where applicable:

- Accession codes, unique identifiers, or web links for publicly available datasets
- A description of any restrictions on data availability
- For clinical datasets or third party data, please ensure that the statement adheres to our policy

Source data underlying Figures 2–5 are available in Zenodo under accession code 10.5281/zenodo.19636012. Figure 1 is a schematic illustration and has no associated source data. All other data supporting the findings of this study are available within the paper and its Supplementary Information. Details of the MD simulation methods are provided in the Supplementary Methods.Source data underlying Figures 2–5 are available in Zenodo under accession code 10.5281/

zenodo.19636012. Figure 1 is a schematic illustration and has no associated source data. All other data supporting the findings of this study are available within the paper and its Supplementary Information. Details of the MD simulation methods are provided in the Supplementary Methods.

Research involving human participants, their data, or biological material

Policy information about studies with human participants or human data. See also policy information about sex, gender (identity/presentation), and sexual orientation and race, ethnicity and racism.

Reporting on sex and gender	The study did not involve human research participants.
Reporting on race, ethnicity, or other socially relevant groupings	The study did not involve human research participants.
Population characteristics	The study did not involve human research participants.
Recruitment	The study did not involve human research participants.
Ethics oversight	The study did not involve human research participants.

Note that full information on the approval of the study protocol must also be provided in the manuscript.

Field-specific reporting

Please select the one below that is the best fit for your research. If you are not sure, read the appropriate sections before making your selection.

☒ Life sciences ☐ Behavioural & social sciences ☐ Ecological, evolutionary & environmental sciences

For a reference copy of the document with all sections, see nature.com/documents/nr-reporting-summary-flat.pdf

Life sciences study design

All studies must disclose on these points even when the disclosure is negative.

Sample size	No power analysis on sample size was performed. Sample sizes are clearly reported in the figure legends. Sample sizes were determined by allowable error size and accuracy, as well as resources (generally n>=3). Two-way analysis of variance (ANOVA) and Šídák's multiple comparisons or one-way ANOVA and Dunn's multiple comparisons were used when comparing the groups.
Data exclusions	No data was excluded.
Replication	All experiments were successfully repeated at least 2 times to confirm the reproducibility
Randomization	No animal studies were included in the study. For in vitro cell experiments, the cells were treated with samples randomly with at least 3 replicate wells, without selecting areas of treatments.
Blinding	Investigators were blinded during flow cytometry data analysis, where only well names were shown before completing analysis of the results.

Reporting for specific materials, systems and methods

We require information from authors about some types of materials, experimental systems and methods used in many studies. Here, indicate whether each material, system or method listed is relevant to your study. If you are not sure if a list item applies to your research, read the appropriate section before selecting a response.

Materials & experimental systems

n/a	Involved in the study	
☒	☐	Antibodies
☐	☒	Eukaryotic cell lines
☒	☐	Palaeontology and archaeology
☒	☐	Animals and other organisms
☒	☐	Clinical data
☒	☐	Dual use research of concern
☒	☐	Plants

Methods

n/a	Involved in the study	
☒	☐	ChIP-seq
☐	☒	Flow cytometry
☒	☐	MRI-based neuroimaging

Eukaryotic cell lines

Policy information about cell lines and Sex and Gender in Research

Cell line source(s)	HEK293T cell line was obtained from ATCC (CRL-3216), HEK293F cell line was obtained from ThermoFisher Scientific (R79007). These cells are isolated from human embryonic kidneys (HEK).
Authentication	The cell lines were authenticated by morphology.
Mycoplasma contamination	The cell lines tested negative for mycoplasma contamination.
Commonly misidentified lines (See ICLAC register)	No commonly misidentified cell lines were used in the study.

Plants

Seed stocks	No plants were used in the study.
Novel plant genotypes	No plants were used in the study.
Authentication	No plants were used in the study.

Flow Cytometry

Plots

Confirm that:

☒ The axis labels state the marker and fluorochrome used (e.g. CD4-FITC).

☒ The axis scales are clearly visible. Include numbers along axes only for bottom left plot of group (a 'group' is an analysis of identical markers).

☒ All plots are contour plots with outliers or pseudocolor plots.

☒ A numerical value for number of cells or percentage (with statistics) is provided.

Methodology

Sample preparation	The adherent cells were digested from the plates with 0.25% Trypsin, the suspension cells were collected from the plates. All cells were centrifuges and resuspended in PBS before loading the samples on Attune™ NxT Flow Cytometer.
Instrument	ThermoFIsher Scientific, Attune™ NxT Flow Cytometer.
Software	FlowJo (v.10.8.1) was used to analyze the data.
Cell population abundance	10,000-50,000 cells were collected from each data point.
Gating strategy	Gating was first based on FSC/SSC together with FSC-A/FSC-H (singlet populations). The cell populations within the gate were further analyzed based on expression of targeted protein. Untreated and unstained groups were used to determine the positive.

☒ Tick this box to confirm that a figure exemplifying the gating strategy is provided in the Supplementary Information.
