# Supplementary material for: Trivalent ions kinetic-gating for producing high-concentration and shelf-stable plasmid DNA/PEI particles
Source: Nat Commun. 2026 Jun 2;17:7107. doi: 10.1038/s41467-026-73921-4 (PMC13392119; doi:10.1038/s41467-026-73921-4)
Supplement: Supplementary file 3 — Transparent Peer Review file [file 41467_2026_73921_MOESM3_ESM.pdf]

nature portfolio

Peer Review File

Trivalent Ions Kinetic-Gating for Producing High-Concentration and Shelf-Stable Plasmid DNA/PEI Particles

Corresponding Author: Professor Hai-Quan Mao

This file contains all reviewer reports in order by version, followed by all author rebuttals in order by version.

Version 1:

Reviewer comments:

Reviewer #1

(Remarks to the Author)
This work is an interesting proposal for preparing and storing preformed PEI-pDNA complexes and escalating for AAV production. The novelty of this proposal is the possibility of storing the complexes at -80ºC and then reusing them when thawed without losing their potential.
The use of citric acid to increase the colloidal integrity and transfection efficiency of nanoparticles is not new. In the literature, some papers utilize citric acid to produce more efficient PEI-pDNA particles for transfection processes. Some of them are homogeneous in size and zeta potential and colloidal stable for 4 hours to 24 hours.
It is worth noting that the authors used only a plasmid encoding luciferase (10 w/w) or a single plasmid encoding 100% tdTomato. However, it would have been interesting to use a combination of different plasmids to mimic the AAV assembly components, perhaps plasmids encoding fluorescent proteins. In this case, the authors could have shown the percentage of each plasmid that is incorporated into the cells.
Surprisingly, after preparing a complex with 200 µg/mL, they only use 1µg of DNA per well in transfection studies.
Lines 125-137. I am not sure if the authors are explaining a fact or describing a hypothesis.
Why do the authors focus on 400 nm particles if the 800 nm particles have similar properties?
The simulations used throughout the work seem more hypothetical than real. Are they demonstrated?
Minor comments
Figure 1 is rather confusing. The assembly process flow is not easily followed.
The authors do not indicate which MW of the PEI they are using or the characteristics of this PEI.
Differences between the transfection of PBS and CBS particles are significant. But how many fold?

Reviewer #2

(Remarks to the Author)
This study led by Hu and Mao et al. presents another successful example that translational-driven research can also be fundamentally important. Targeting the practical problem in using pDNA/PEI nanoparticles for transfection especially in large scale, the authors proposed an innovative DNA-PEI assembly approach by incorporating citrate ions to interplay the binding process, where this idea is well-rooted and also strongly supported by in-depth understanding of the DNA-PEI binding fundamentals. Homogeneous mixing of pDNA and PEI prior to particle growth is critical for forming uniform particles. The extended complexation between pDNA and PEI mediated by citrate ions provides sufficient time for the homogeneous mixing to allow particle to grow. This simple approach enabled high-concentration mixing and stabilization of the complex under different storage conditions and manufacturing scales, which is carefully proved as translation-ready.
This manuscript is well organised with sufficient experimental and theoretical support to explore this binding process and following transfection and fabrication. Therefore, I suggest minor revisions by considering the following comments.
1. Interestingly the citrate ions demonstrated to have stronger associations with PEI compared to phosphate ions, enabling near-complete charge neutralization of PEI molecules. Why are citrate ions more effective than phosphate ions in neutralizing the positive charges on PEI?
2. It is hypothesized that “citrate ion as a multivalent and reversible “chelator” to PEI”, where a large number of citrate ions bind with PEI to reduce its positive charge density. However, with a high proportion of citrate ions bind linked to PEI, will the pH of the solution change accordingly? Will the change of pH also influence the binding process?
3. A comprehensive characterization of the particle size along the assembly process has been provided, but how about the

surface charge? What is the surface charge difference when assembled in PBS and CBS?
4. In Figure 2h and I, the label of t=0 and t=4h, freshly prepared and freeze-thawed, cannot be easily identified in the figure, suggest to use color rather than symbol to represent the groups.
5. As shown in Figure 2a and b, it is expected to have a relatively slower growth of particle size for CBS but seems not very clear trend in the data, please provide possible explanations.

Reviewer #3

(Remarks to the Author)
The manuscript presents an innovative kinetic gating strategy for assembling pDNA/PEI particles with improved uniformity and scalability using citrate ions. While the study is well-conceived and the findings are of potential interest, several issues need to be addressed to strengthen the mechanistic insights and experimental support.

Major Comments:

1. The claim of reversible "chelation" between citrate and PEI remains speculative without quantitative binding data. It is essential to provide Isothermal Titration Calorimetry (ITC) data to determine the binding constants (Kd) for citrate-PEI, PEI-pDNA, and citrate-PEI-pDNA interactions. A comparative analysis of these values is needed to clarify their distinctions and underlying relationships.

2. The study focuses exclusively on citrate ions. To evaluate whether the observed effects are unique to trivalent citrate, control experiments with other multivalent ions, such as divalent (e.g., SO_4^{2-}) or alternative trivalent ions, should be included.

3. The figure organization could be improved for clarity. It is recommended to re-group the panels to more clearly separate mechanistic studies (e.g., MD simulations), assembly kinetics, and functional outcomes (e.g., transfection efficiency).

4. For Figures 2h and 2i, please use distinct bar colors or patterns to differentiate the experimental groups (e.g., PBS vs. CBS, different particle sizes). The current grayscale scheme may hinder readability.

5. For Figure 2f (TEM images), please provide additional, in-focus TEM fields of view to better represent particle morphology and uniformity. The current images appear limited in scope and focus.

6. Data on nanoparticle cytotoxicity is missing from the manuscript. This is a critical component for assessing the clinical translation potential of any novel transfection reagent. Standard cell viability assays should be performed.

7. The stability of the particles under physiologically relevant conditions is not evaluated. The particles should be incubated in cell culture media, both with and without 10% FBS, at 37°C. Changes in particle size (by DLS) and PDI should be monitored over a relevant time course. Serum protein adsorption can lead to aggregation or opsonization, which would severely limit practical application.

Version 2:

Reviewer comments:

Reviewer #1

(Remarks to the Author)
The authors have answered all my concerns and this manuscript is now accepted for publication

Reviewer #2

(Remarks to the Author)
The authors have well addressed all my comments and revised the manuscript accordingly to further improve the quality of this work, thus it is recommended to accept as it is.

Reviewer #3

(Remarks to the Author)
I would like to thank the authors for their thoughtful and thorough responses to all of my previous concerns. The additional experiments, including the ITC binding studies, control experiments with multivalent ions, cell viability assays, stability tests under physiological conditions, and histopathological analyses, have substantially strengthened the manuscript. The revisions to the figures and the clarification of mechanistic points have also improved the clarity and readability of the work. I am satisfied that all of my comments have been adequately addressed. Therefore, I recommend that the manuscript be accepted in its current form.

Open Access This Peer Review File is licensed under a Creative Commons Attribution 4.0 International License, which permits use, sharing, adaptation, distribution and reproduction in any medium or format, as long as you give appropriate credit to the original author(s) and the source, provide a link to the Creative Commons license, and indicate if changes were made.
In cases where reviewers are anonymous, credit should be given to 'Anonymous Referee' and the source.
The images or other third party material in this Peer Review File are included in the article's Creative Commons license, unless indicated otherwise in a credit line to the material. If material is not included in the article's Creative Commons license and your intended use is not permitted by statutory regulation or exceeds the permitted use, you will need to obtain permission directly from the copyright holder.
To view a copy of this license, visit https://creativecommons.org/licenses/by/4.0/

Responses to Reviewers' Comments

General response:

We thank the reviewers for their thorough reviews and suggestions. We have carefully considered all the comments in our revision effort. As a result of the revision, we have revised **Figs. 1–3,** and added **Supplementary Figs. 1–3, 6, 8–11**. These revisions have significantly improved the quality of our manuscripts. The point-by-point responses are appended below.

Reviewer #1:

This work is an interesting proposal for preparing and storing preformed PEI-pDNA complexes and escalating for AAV production. The novelty of this proposal is the possibility of storing the complexes at -80ºC and then reusing them when thawed without losing their potential.

1. The use of citric acid to increase the colloidal integrity and transfection efficiency of nanoparticles is not new. In the literature, some papers utilize citric acid to produce more efficient PEI-pDNA particles for transfection processes. Some of them are homogeneous in size and zeta potential and colloidal stable for 4 hours to 24 hours.

[Response]: We appreciate the reviewer's careful literature review and agree that citrate has been explored previously as a formulation additive for PEI–pDNA complexes. Several representative studies are worth noting: low molecular weight (MW) PEI–citrate polyplexes that improved in vitro transfection (Girrón-González *et al.*, *Bioconjugate Chem.* 2016); a citric-acid-based polyester elastomer as a scaffold to localize PEI/DNA polyplexes and achieve prolonged transgene expression (Zhang *et al.*, *Biomaterials* 2009); and very recent uses of citrate or borate to moderate PEI toxicity (Suner *et al., Biomedicines* 2024; Eberhardy *et al., Cytotechnology* 2009) or to create 90-nm PEI–citrate "nanocores" for antisense oligonucleotide delivery (de la Hoz *et al., Biomolecules* 2022).

Those important precedent studies, however, **stopped at empirical buffer optimization**. None reported a route to **size-controlled sub-micron particles, at >10-fold higher DNA concentration, week-scale stability, or industrial bioreactor validation**. Our work advances the field in three keyways:

- **Kinetic-gating mechanism**. We use citrate as a *transient* trivalent counterion that slows the initial PEI–DNA complexation rate, decouples nucleation from growth, and yields uniform nuclei that can be predictably expanded. This mechanistic control, supported by MD simulations and ITC data, differs fundamentally from prior single-composition screens.
- **Process intensification**. The kinetic gating allows one-step assembly of particles containing ≥200 µg/mL plasmid (10× conventional protocols) while maintaining a narrow size distribution and full transfection potency after freeze-thaw, and a high degree of on-bench stability at ambient temperature.
- **Scalability and validation.** We demonstrate seamless transfer of the formulation to a 20-L equivalent bioreactor and show that the particles match or exceed the best-in-class industrial benchmark in AAV production, confirming practical relevance beyond small-scale cell culture.

Taken together, our contribution is not the mere inclusion of citrate, but the establishment of a general, kinetics-driven design principle that links multivalent ion regulation to scalable manufacturing outcomes.

We have clarified this distinction in the revised manuscript (**Introduction, lines 69–76; Discussion, lines 391–395, 414–416**) and now cite all relevant prior reports to highlight what is genuinely new in the present study.

Page 3, line 69:

Formulation studies have explored buffer additives to moderate PEI–pDNA interactions. Notably, trivalent citrate ions have been used as a *formulation excipient* to enhance *in vitro* transfection or to extend bench stability to a few hours. Representative studies include low-molecular-weight PEI–citrate polyplexes that improved luciferase expression in B16-F10 cells[21], citric-acid-based polyester elastomer as a scaffold for pDNA/PEI complexes[22], and recent reports that citrate reduces PEI cytotoxicity while conferring 4–24 h colloidal stability[23-25]. These contributions demonstrate the feasibility of introducing multivalent anions, yet they all employed empirically selected citrate concentrations and did not elucidate the underlying rate processes.

Page 14, line 391:

Our findings establish kinetic gating by multivalent counterions as a general principle for assembling polyelectrolyte nanocomplexes under manufacturing-relevant conditions. Where earlier citrate studies provided useful but short-lived stability at dilute DNA loadings, we show that modulating the early-stage complexation rate unlocks tenfold higher plasmid concentration, high room-temperature stability, and full stability after cryo-storage.

Page 14, line 414:

Together, these advances convert multivalent ions such as citrate into an engineered kinetic modulator, offering both a practical solution to current manufacturing bottlenecks and a framework for future ion-mediated assembly strategies.

References:

21. Girón-González, M. D. et al. Polyelectrolyte complexes of low molecular weight PEI and citric acid as efficient and nontoxic vectors for in vitro and in vivo gene delivery. *Bioconjugate Chem.* 27, 1803–1812 (2016).
22. Zhang, X.-Q. et al. Sustained transgene expression via citric acid-based polyester elastomers. *Biomaterials* 30, 2632–2641 (2009).
23. Suner, S. S., Ayyala, R. S. & Sahiner, N. Antipathogenic Activity of Betainized Polyethyleneimine Sprays Without Toxicity. *Biomedicines* 12, 2462 (2024).
24. Eberhardy, S. R., Radzniak, L. & Liu, Z. Iron (III) citrate inhibits polyethylenimine-mediated transient transfection of Chinese hamster ovary cells in serum-free medium. *Cytotechnology* 60, 1–9 (2009).
25. de la Hoz, R., Diban, N., Berciano, M. T., San Emeterio, C., Urtiaga, A., Lafarga, M., Rodríguez-Rey, J. C. & Tapia, O. Coaxial Synthesis of PEI-Based Nanocarriers of Encapsulated RNA-Therapeutics to Specifically Target Muscle Cells. *Biomolecules* 12, 1012 (2022).

2. It is worth noting that the authors used only a plasmid encoding luciferase (10 w/w) or a single plasmid encoding 100% tdTomato. However, it would have been interesting to use a combination of different plasmids to mimic the AAV assembly components, perhaps plasmids encoding fluorescent proteins. In this case, the authors could have shown the percentage of each plasmid that is incorporated into the cells.

[Response]: We agree that co-delivery of multiple plasmids is highly relevant to AAV assembly systems, where coordinated intracellular delivery and expression of helper, rep/cap, and transgene plasmids within the same producer cell are essential for efficient viral production.

To directly examine multi-plasmid delivery capability, we performed additional co-transfection experiments using defined input ratios of GFP- and tdTomato-encoding plasmids formulated using our citrate-mediated kinetic control strategy. Single-cell fluorescence was quantified by flow cytometry to determine GFP^{+} and $tdTomato^{+}$ cell populations. As the plasmid mixing ratio was varied, we observed a composition-dependent shift in reporter-positive populations with a near-linear trend across tested conditions (**Supplementary Fig. 10**). Although the percentage of positive cells did not exactly match the theoretical input ratio, likely due to stochastic uptake, intracellular trafficking variability, and expression threshold effects related to the two different plasmids, no preferential enrichment of one plasmid over the other was detected. These data indicate that the formulation method does not introduce selective encapsulation or delivery bias in multi-plasmid systems.

Importantly, the later sections of the manuscript include functional AAV production data obtained in collaboration with industry partners using the standard multi-plasmid packaging system. In addition to viral genome titers, genome titer-to-capsid titer ratios were quantified (**Supplementary Fig. 11**). This ratio serves as an indicator of coordinated intracellular delivery and expression of packaging components, as imbalanced plasmid delivery typically reduces genome incorporation efficiency. Across multiple formulation concentrations, citrate-mediated PEI–pDNA complexes yielded genome-to-capsid ratios comparable to or exceeding those of the industry control formulation. These results provide functional validation that our formulation strategy supports balanced co-delivery of the required plasmids for AAV assembly. If substantial stoichiometric bias or selective plasmid incorporation were occurring, significant reductions in viral yield and/or altered genome-to-capsid ratios would be expected.

Collectively, the dual-reporter experiments, together with the AAV production and genome-to-capsid ratio data, demonstrate that our citrate-mediated kinetic assembly approach enables reliable, compositionally balanced multi-plasmid delivery relevant to AAV packaging. We have included the additional data in the revised manuscript (**Supplementary Figs. 10 and 11**) and clarified this point in the Results section.

Page 13, line 361:

Furthermore, to evaluate multi-plasmid delivery capability, GFP- and tdTomato-encoding plasmids were co-formulated at defined input ratios using the 400-nm CBS-mediated nanoparticles. Single-cell fluorescence analysis by flow cytometry revealed a composition-dependent shift in the proportions of GFP^{+} and $tdTomato^{+}$ cells as the input plasmid ratio was varied (**Supplementary Fig. 10**), showing a near-linear trend without preferential enrichment of either plasmid. These results indicate that the citrate-mediated kinetic assembly strategy does not introduce selective encapsulation or delivery bias in multi-plasmid systems.

Page 13, line 381:

In addition to viral genome titers, genome-to-capsid titer ratios were quantified as an indicator of coordinated intracellular delivery and expression of packaging plasmids. Across multiple formulation concentrations, citrate-mediated PEI–pDNA complexes yielded genome-to-capsid ratios comparable to or exceeding those of the industry control, supporting balanced multi-plasmid delivery during AAV assembly (**Supplementary Fig. 11**).

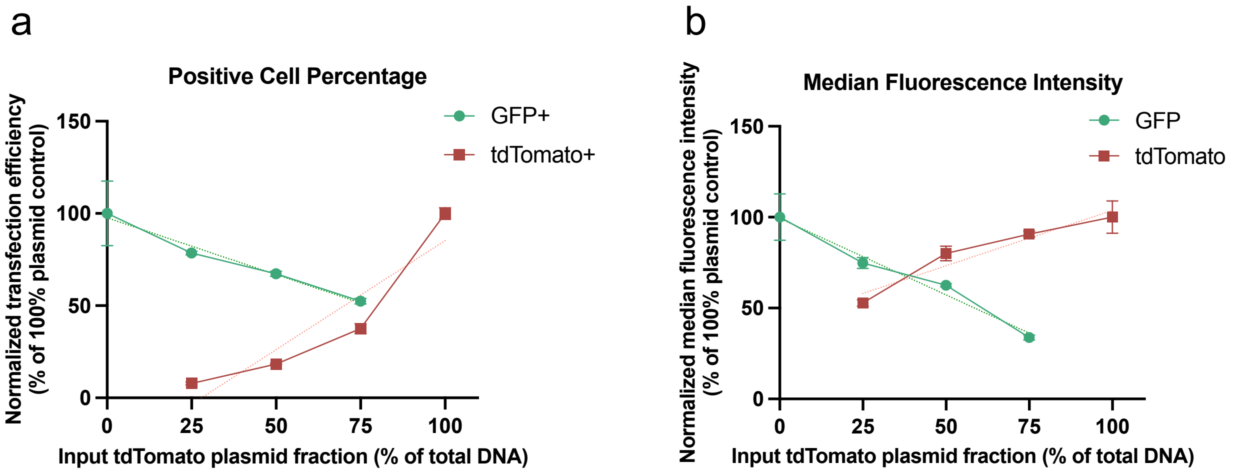

Supplementary Fig. 10. Multi-plasmid co-delivery performance of citrate-mediated PEI–pDNA nanoparticles. (**a**) Percentage of GFP$^+$ and tdTomato$^+$ cells following co-transfection with defined input ratios of GFP- and tdTomato-encoding plasmids formulated using 400-nm CBS-mediated nanoparticles. (**b**) Mean fluorescence intensity (MFI) of GFP and tdTomato signals under the same plasmid mixing conditions. Data are presented as mean ± s.d. (n = 3).

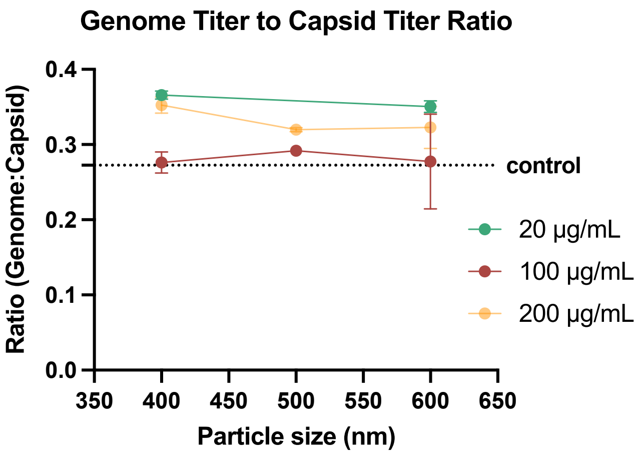

Supplementary Fig. 11. Genome-to-capsid ratio analysis of AAV produced using citrate-mediated PEI–pDNA complexes. Genome titer and capsid titer were quantified to determine the genome-to-capsid ratio as an indicator of coordinated multi-plasmid delivery during AAV assembly.

3. Surprisingly, after preparing a complex with 200 µg/mL, they only use 1µg of DNA per well in transfection studies.

[Response]: The preparation concentration of 200 µg/mL was intentionally selected to enable a high-concentration formulation suitable for large-scale applications, particularly in bioreactor settings where minimizing dosing volume is critical. In industrial-scale transfection processes, adding large volumes of dilute PEI–pDNA complexes (e.g., ~5% of total culture volume) can be time-consuming and operationally challenging. Therefore, developing a high-concentration, stable formulation is an important practical objective of this study.

In contrast, the DNA dose used in the in vitro transfection assays (1 µg per well) was chosen based on established cellular tolerance limits and standard benchmarking conditions for the relevant cell lines. Using a biologically appropriate dose ensures meaningful comparison with conventional transfection protocols and avoids confounding effects associated with DNA overdosing. Importantly, the ability to prepare PEI–pDNA particles at high bulk concentration does not necessitate high dosing in vitro; rather, it provides advantages in scalability, storage, and handling while allowing precise aliquoting to application-relevant concentrations.

4. Lines 125-137. I am not sure if the authors are explaining a fact or describing a hypothesis.

[Response]: We agree that the original text did not clearly distinguish between experimentally supported findings and mechanistic hypotheses. In the revised manuscript, we have clarified this section by explicitly separating the proposed kinetic-gating strategy and its experimental outcomes from the mechanistic hypothesis describing citrate–PEI interactions.

Specifically, statements supported by experimental data, such as the observed improved uniformity, are now presented as results. In contrast, the proposed mechanism by which citrate transiently associates with PEI to reduce effective charge density and modulate complexation kinetics is clearly identified as a hypothesis intended to rationalize the observed behavior. This revision improves clarity and avoids conflating interpretation with established fact.

Page 5, Line 137:

Here, we propose a new trivalent ion-mediated kinetic gating method to substantially slow down the rate of PECn, thus increasing τ_c to match the slower mixing process, thereby improving the uniformity of the PECn. Experimentally, we observe that CBS reduces particle polydispersity and enhances size control across mixing regimes. We hypothesize that citrate functions as a multivalent, reversible chelator of PEI. Due to its small molecular weight and high diffusivity, citrate can rapidly associate with protonated amines on PEI during mixing, transiently reducing the effective positive charge density. This reduction likely decreases the energetic driving force for immediate PEI–pDNA complexation, thereby slowing PEC formation. At higher pDNA concentrations, where τ_c is intrinsically shorter, the gating effect becomes more pronounced (**Fig. 1b**). Given the higher charge density of pDNA, citrate is expected to be displaced during final PEC formation, restoring strong PEI–DNA interactions. We therefore propose that citrate-mediated kinetic modulation enables PEC formation under conditions where τ_c approaches or exceeds τ_m (**Fig. 1b**), reducing sensitivity to mixing rate and improving uniformity across scales.

5. Why do the authors focus on 400 nm particles if the 800 nm particles have similar properties?

[Response]: While ~800 nm particles exhibit comparable colloidal stability and, in some cases, similar transfection efficiency, we focused on ~400 nm particles because this size range represents the optimized window for transfection performance based on both prior literature and our own experimental validation. Specifically, Hu et al. (Nano Lett., 2021) demonstrated that PEI–DNA complexes in the ~300–500 nm range achieve superior transfection efficiency compared to significantly larger aggregates, consistent with size-dependent cellular uptake mechanisms. In our study, this trend is also supported by the data presented in **Fig. 5**, where particles around 400 nm consistently delivered robust transfection outcomes on the tested cell lines.

In addition, although 800 nm particles can perform comparably under certain conditions, we observed that larger particles exhibit more pronounced increases in polydispersity index (PDI) and greater variability in batch reproducibility. Larger particles also exhibit a greater tendency toward sedimentation and aggregation during handling, and, in some cases, increased cytotoxicity relative to the 400 nm formulations. Thus, while 800 nm particles remain functionally viable, ~400 nm represents a more reproducible and biologically favorable operational window.

Focusing on ~400 nm particles, therefore, allowed us to demonstrate that the citrate-mediated kinetic control strategy reliably accesses an optimized transfection size regime rather than merely producing large aggregates with acceptable performance.

6. The simulations used throughout the work seem more hypothetical than real. Are they demonstrated?

[Response]: We would like to clarify that the simulations in this manuscript are not hypothetical illustrations but fully implemented coarse-grained molecular dynamics (MD) simulations designed to capture the fundamental physicochemical behavior of PEI–DNA complexes under our experimental conditions.

Figure 3e–g presents the differences in PEI charge states in PBS and CBS solutions. These data are obtained directly from MD trajectories using a chemically detailed coarse-grained model with explicit electrostatics. The model incorporates the full chemical structure of all relevant components, including PEI, citrate, sodium, and chloride ions, using the MARTINI force field. Importantly, the simulations demonstrate and explain key experimental observations, including the reduced zeta potential in CBS-formulated complexes.

Figure 3h–i compares PEI–DNA interaction energies between PBS and CBS. The simulations show that PEI–DNA binding is significantly stronger in PBS, leading to kinetically trapped, heterogeneous complexes, explaining our experimental measurements. In contrast, citrate buffers weaken PEI–DNA interactions and promote the formation of more homogeneous and colloidally stable particles. The model again incorporates the complete chemical structures of PEI, DNA, citrate, sodium, and chloride ions. The DNA used in the simulations was a full 24-bp sequence (dCdGdCdGdAdAdTdTdCdGdCdGdCdGdCdGdAdAdTdTdCdGdCdG), demonstrating structural and electrostatic accuracy rather than generic modeling. Additionally, the binding free-energy profiles shown in **Figure 3h–i** were obtained using umbrella sampling MD simulations, a

well-established and highly accurate technique for quantifying molecular binding thermodynamics.

We would also like to highlight that the simulation results presented in the Supplementary Information are likewise not hypothetical. Because these figures examine larger length-scale behavior, we used a bead-spring coarse-grained model to efficiently capture mesoscale assembly. Although simplified, this model retains essential electrostatic features, including the effects of trivalent and divalent anions, and therefore provides physically meaningful insights into the stability and aggregation tendencies under different buffer conditions.

Minor comments

1. Figure 1 is rather confusing. The assembly process flow is not easily followed.

[Response]: We have substantially revised Figure 1 to improve the visual flow and clarify the sequence of events in the assembly process.

The updated figure now clearly illustrates the two key stages involved in particle formation: the initial polyelectrolyte complexation (PECn) between pDNA and PEI and the subsequent nanoparticle assembly (NPa) process that leads to the formation of 400–600 nm transfection particles (**Fig. 1a**). In addition, the particle growth trajectory is now divided into three size regimes (P1, P2, and P3) to better illustrate the progression of particle formation.

Panel (b) has also been revised to more clearly depict the relative time scales of the mixing process (τ_m), the PEI–pDNA complexation process (τ_c), and the nanoparticle assembly process (τ_a). This schematic highlights the key challenge addressed in this study: under conventional conditions, the complexation time (τ_c) is substantially shorter than the mixing time (τ_m), which can lead to heterogeneous PEC formation. The citrate-mediated kinetic gating strategy proposed in this work increases the effective τ_c, bringing it closer to the mixing time scale and thereby improving the uniformity of the resulting nanoparticles.

We believe these revisions make the conceptual framework and assembly sequence illustrated in **Figure 1** substantially clearer and easier to follow.

2. The authors do not indicate which MW of the PEI they are using or the characteristics of this PEI.

[Response]: In this study, we used PEIpro® (Polyplus Sartorius, France), supplied as a commercial transfection-grade PEI solution at 1 mg/mL. The manufacturer does not disclose the exact molecular weight distribution of PEIpro®, as it is a proprietary linear polyethyleneimine formulation optimized for high-efficiency DNA transfection. However, according to the product documentation, PEIpro® is a linear PEI-based reagent with a defined, reproducible polymer composition, designed for large-scale transfection applications.

While the precise molecular weight distribution is not publicly specified, all experiments were performed using the same lot and concentration (1 mg/mL stock), ensuring internal consistency throughout the study. Importantly, our mechanistic conclusions focus on citrate-mediated modulation of PEI–DNA complexation kinetics rather than on intrinsic molecular-weight-dependent effects. Therefore, although the exact MW distribution is not provided by the

manufacturer, the reproducibility of our results across multiple experiments supports the robustness of the reported findings.

We have clarified in the Methods section that PEIpro® (Polyplus Sartorius, France) was used as received.

Page 15, Line 422:

Poly(ethyleneimine) (PEIpro, Polyplus Sartorius, France) was similarly dissolved at 580.8 µg/mL in a PEI dilution buffer with 19% (w/w) trehalose.

3. Differences between the transfection of PBS and CBS particles are significant. But how many fold?

[Response]: As shown in **Fig. 2i**, under freshly prepared conditions, PEI–pDNA particles formulated in CBS exhibited a ~2-fold increase in transfection efficiency compared to those prepared in PBS. After a freeze–thaw cycle, the difference became more pronounced, with CBS-formulated particles demonstrating a ~5-fold higher transfection efficiency relative to PBS.

These results indicate that while CBS provides a moderate enhancement under fresh conditions, its benefit becomes substantially amplified following freeze–thaw stress, highlighting the improved structural robustness and functional stability conferred by citrate-mediated kinetic control.

We have now included these fold-change values explicitly in the revised manuscript for clarity.

Page 8, line 213:

More importantly, when we encapsulated luciferase pDNA in the particles and transfected HEK293T cells using stabilized particles, 400-nm pDNA/PEI particles assembled in CBS showed a significantly higher transfection efficiency than those prepared in PBS (**Fig. 2i**), ~2-fold on freshly prepared particles and ~5-fold on freeze-thawed particles, presumably due to the more uniform size distribution within the optimal size range of 400 to 600 nm.

Reviewer #2:
This study led by Hu and Mao et al. presents another successful example that translational-driven research can also be fundamentally important. Targeting the practical problem in using pDNA/PEI nanoparticles for transfection especially in large scale, the authors proposed an innovative DNA-PEI assembly approach by incorporating citrate ions to interplay the binding process, where this idea is well-rooted and also strongly supported by in-depth understanding of the DNA-PEI binding fundamentals. Homogeneous mixing of pDNA and PEI prior to particle growth is critical for forming uniform particles. The extended complexation between pDNA and PEI mediated by citrate ions provides sufficient time for the homogeneous mixing to allow particle to grow. This simple approach enabled high-concentration mixing and stabilization of the complex under different storage conditions and manufacturing scales, which is carefully proved as translation-ready.

This manuscript is well organised with sufficient experimental and theoretical support to explore this binding process and following transfection and fabrication. Therefore, I suggest minor revisions by considering the following comments.

1. Interestingly the citrate ions demonstrated to have stronger associations with PEI compared to phosphate ions, enabling near-complete charge neutralization of PEI molecules. Why are citrate ions more effective than phosphate ions in neutralizing the positive charges on PEI?

[Response]: We thank the reviewer for this insightful question. Multivalent counterion theory predicts that higher-valency anions exhibit disproportionately stronger electrostatic association with oppositely charged polyelectrolytes due to electrostatic correlation effects and cooperative condensation mechanisms (Bloomfield, 1997; Rouzina & Bloomfield, 1996). Under our formulation conditions, citrate predominantly exists as a trivalent anion ($Citrate^{3-}$), enabling a single citrate molecule to associate with multiple protonated amines along the PEI backbone simultaneously. In contrast, phosphates act as monovalent and divalent ions at pH 7 because they exist predominantly as $H_2PO_4^-$ and HPO_4^{2-}.

Furthermore, citrate contains three carboxylate groups arranged on a flexible backbone, allowing multidentate binding and potential bridging interactions between adjacent PEI chains, a phenomenon well documented for multivalent counterions in polyelectrolyte systems (Bordi et al., 2004). Previous studies have directly demonstrated citrate-mediated modulation of PEI–DNA complexes (Girrón-González et al., 2016), supporting the ability of citrate to reduce effective PEI charge density.

Collectively, these physicochemical principles explain why citrate more efficiently condenses PEI and enables near-complete charge neutralization compared to phosphate under comparable ionic strength conditions.

Relevant References:

Bloomfield, V. A. Condensation of DNA by multivalent cations: considerations on mechanism. Biopolymers 44, 269–282 (1997).

Rouzina, I. & Bloomfield, V. A. Competitive electrostatic binding of charged ligands to polyelectrolytes. J. Phys. Chem. 100, 9977–9989 (1996).

Bordi, F., Cametti, C. & Sennato, S. Electrostatic interactions in polyelectrolyte solutions: multivalent counterion effects. J. Phys.: Condens. Matter 16, R1423–R1463 (2004).

Girrón-González, M. D. et al. Polyelectrolyte complexes of low molecular weight PEI and citric acid as efficient and nontoxic vectors for in vitro and in vivo gene delivery. Bioconjug. Chem. 27, 1803–1812 (2016).

2. It is hypothesized that "citrate ion as a multivalent and reversible "chelator" to PEI", where a large number of citrate ions bind with PEI to reduce its positive charge density. However, with a high proportion of citrate ions bind linked to PEI, will the pH of the solution change accordingly? Will the change of pH also influence the binding process?

[Response]: We thank the reviewer for raising this important point. We agree that pH plays a role in PEI–DNA complexation. In our system, the bulk pH decreases after mixing, reaching approximately 4.58 in CBS-containing solution and 4.15 in PBS. This pH drop arises from acid–base equilibration during polyelectrolyte complex formation. PEI contains a high density of amine groups with broad pKa values (~4–10), and upon interaction with DNA phosphates and buffer counterions, the protonation equilibria are redistributed. Nonetheless, the difference in the ionization degree of PEI between pH 4.15 and pH 4.85 is small, given its apparent pH range of 8–10.

We acknowledge that such pH changes influence the protonation state of PEI and therefore affect electrostatic interactions. However, the system is not governed by pH alone. PEI–DNA assembly reflects a combined effects of (i) pH-dependent PEI protonation, (ii) ionic strength, (iii) multivalent ion binding geometry, and (iv) the relative concentrations of PEI and DNA during complexation (**Supplementary Fig. 5**). Notably, despite PBS reaching a lower pH than the citrate-containing system, citrate ions exhibit stronger effective charge modulation and more controlled complexation behavior. This indicates that the multivalent nature of citrate continues to exert its effects within the system even under acidic conditions.

As a trivalent and multidentate anion, citrate can simultaneously associate with multiple protonated amines on PEI, enabling cooperative charge compensation and transient bridging interactions that mono- or divalent phosphate species cannot provide. Thus, while pH contributes to the overall electrostatic landscape, the enhanced regulation observed in CBS arises from the combined effects of multivalency and ionic mediation rather than from pH change alone.

3. A comprehensive characterization of the particle size along the assembly process has been provided, but how about the surface charge? What is the surface charge difference when assembled in PBS and CBS?

[Response]: We thank the reviewer for this question. We have now measured the zeta potential evolution during assembly using freshly prepared samples. In PBS, the zeta potential increased from ~31–33 mV to ~40–42 mV within ~5–8 min and then remained stable (~39–42 mV) over the remainder of the measurement window. In CBS, the zeta potential started slightly lower (~28–30 mV) and increased to a stable plateau of ~35–37 mV by ~10–12 min. Overall, CBS particles exhibit a consistently lower steady-state zeta potential than PBS by ~4–6 mV, indicating stronger effective charge compensation in citrate-containing buffer. We interpret the initial increase in zeta potential under both conditions as reflecting rapid PEI adsorption and surface presentation during early nucleation, followed by stabilization as complexes mature. The lower

plateau in CBS is consistent with multivalent citrate association, providing more effective screening/neutralization of protonated PEI compared with phosphate species.

We have added these results to the revised manuscript (**Supplementary Fig. 1**) and clarified this point in the Results section.

Page 7, line 183:

The surface charge during assembly was also monitored using zeta potential measurements (**Supplementary Fig. 1**). At steady state, particles assembled in PBS exhibited a zeta potential of ~40–42 mV, whereas CBS-assembled particles reached a slightly lower plateau of ~35–37 mV, consistently displayed a modest reduction (~4–6 mV) in surface charge compared with PBS. This difference in steady-state electrostatic characteristics is consistent with altered ion–polymer interactions during assembly and is further examined in the mechanistic analysis later.

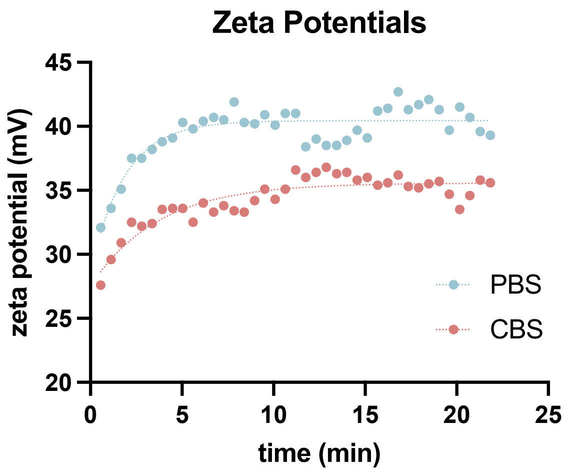

Supplementary Fig. 1. Evolution of zeta potential during PEI–pDNA particle assembly in PBS and CBS. Time-resolved zeta potential measurements of particles assembled in PBS and CBS, monitored immediately after mixing.

4. In Figure 2h and I, the label of t=0 and t=4h, freshly prepared and freeze-thawed, cannot be easily identified in the figure, suggest to use color rather than symbol to represent the groups.

[Response]: In response, we have revised Figures 2h and 2i so that the different conditions (t = 0 and t = 4 h; freshly prepared and freeze–thawed) are now distinguished using colors rather than symbols. The updated color-coded legend improves the clarity and allows the different groups to be more easily identified in the figure.

5. As shown in Figure 2a and b, it is expected to have a relatively slower growth of particle size for CBS but seems not very clear trend in the data, please provide possible explanations.

[Response]: We agree that, under our proposed kinetic-gating framework, citrate-containing buffer (CBS) is expected to reduce the intrinsic PEI–pDNA complexation rate relative to PBS.

However, the growth trends in **Fig. 2a,b** appear similar because the DLS readout reflects the combined outcome of multiple coupled processes (nucleation, growth, aggregation, and compaction) rather than directly measuring the earliest complexation kinetics. In particular, the citrate-mediated “gating” effect is expected to occur during the initial contact/nucleation period (seconds). In contrast, DLS measurements represent ensemble-averaged hydrodynamic diameters over longer timescales (minutes), which can obscure short-timescale kinetic differences.

Additionally, citrate can exert two competing effects: it can transiently reduce the effective PEI charge density to slow the initial complexation step, while also providing stronger electrostatic screening during post-nucleation maturation, which can promote particle growth/compaction once nuclei are formed. As a result, the apparent size-growth curves can converge even though the underlying assembly pathway and population uniformity differ.

Taken together, we interpret **Fig. 2a,b** as showing that CBS provides a broader, more controllable size-control window and reduced sensitivity to mixing conditions, rather than requiring a strictly monotonic reduction in mean growth rate at every time point.

Reviewer #3:

The manuscript presents an innovative kinetic gating strategy for assembling pDNA/PEI particles with improved uniformity and scalability using citrate ions. While the study is well-conceived and the findings are of potential interest, several issues need to be addressed to strengthen the mechanistic insights and experimental support.

Major Comments:

1. The claim of reversible "chelation" between citrate and PEI remains speculative without quantitative binding data. It is essential to provide Isothermal Titration Calorimetry (ITC) data to determine the binding constants (Kd) for citrate-PEI, PEI-pDNA, and citrate-PEI-pDNA interactions. A comparative analysis of these values is needed to clarify their distinctions and underlying relationships.

[Response]: We thank the reviewer for this important suggestion regarding quantitative binding evidence for citrate–PEI interactions. We agree that the term "reversible chelation" describes a mechanistic hypothesis and that direct thermodynamic measurements are valuable. In response, we performed ITC experiments by titrating CBS (citrate-containing buffer) or PBS into PEI (PEIpro®). The resulting thermograms show that citrate-containing injections produce substantially larger heat effects than PBS injections, particularly at low molar ratios, and a distinct integrated-heat profile (**Supplementary Fig. 6**). This indicates a stronger and more cooperative association between citrate and PEI than between phosphate and PEI under comparable conditions.

At the same time, we emphasize that extracting a single binding constant (Kd) for citrate–PEI (and especially for PEI–pDNA or citrate–PEI–pDNA) from ITC is not straightforward in this system. PEI–ion and PEI–DNA interactions are highly multivalent and non-stoichiometric, and the observed heats include contributions from coupled processes such as PEI protonation equilibria, counterion release/redistribution, and (for PEI–DNA) complex growth/aggregation. These effects violate the assumptions of a simple one-site (or few-site) binding model, such that an apparent Kd value derived from standard fitting would not be physically meaningful or reproducible across concentration/ionic conditions.

Therefore, we use ITC here as a comparative thermodynamic readout demonstrating that citrate interacts more strongly with PEI than phosphate, consistent with our proposed kinetic-gating mechanism. Importantly, this conclusion is further supported by independent measurements (e.g., the lower zeta potential in CBS vs PBS during assembly in **Supplementary Fig. 1**), which collectively indicate stronger effective charge compensation by citrate.

Rigorous quantification of apparent association constants for this multicomponent polyelectrolyte system would require a dedicated study to explore various specialized experimental designs (e.g., competition ITC with controlled pH and matched ionic strength, potentiometric titration to quantify PEI protonation shifts, or electrophoretic mobility/charge regulation analysis), as well as theoretical treatment, which is beyond the scope of the current revision.

We have added these results to the revised manuscript (**Supplementary Fig. 6**) and clarified this point in the Results section.

Page 9, line 251:

To experimentally quantify the thermodynamic association between buffer ions and PEI, we performed isothermal titration calorimetry (ITC) by titrating CBS or PBS into PEI solution. Citrate-containing injections produced substantially larger heat changes compared to PBS under matched conditions (**Supplementary Fig. 6**), indicating stronger and more cooperative interactions between citrate and protonated PEI. The integrated heat profile further supports a more significant enthalpic contribution in the citrate system, consistent with multivalent ion pairing and enhanced charge compensation. Due to the multivalent and non-stoichiometric nature of PEI–ion interactions, extraction of a single binding constant is not physically meaningful; the observed heat includes coupled contributions from protonation equilibria and counterion redistribution. Nevertheless, the comparative ITC signatures provide direct thermodynamic evidence that citrate associates more strongly with PEI than phosphate species, supporting the mechanistic interpretation derived from MD and PALS measurements.

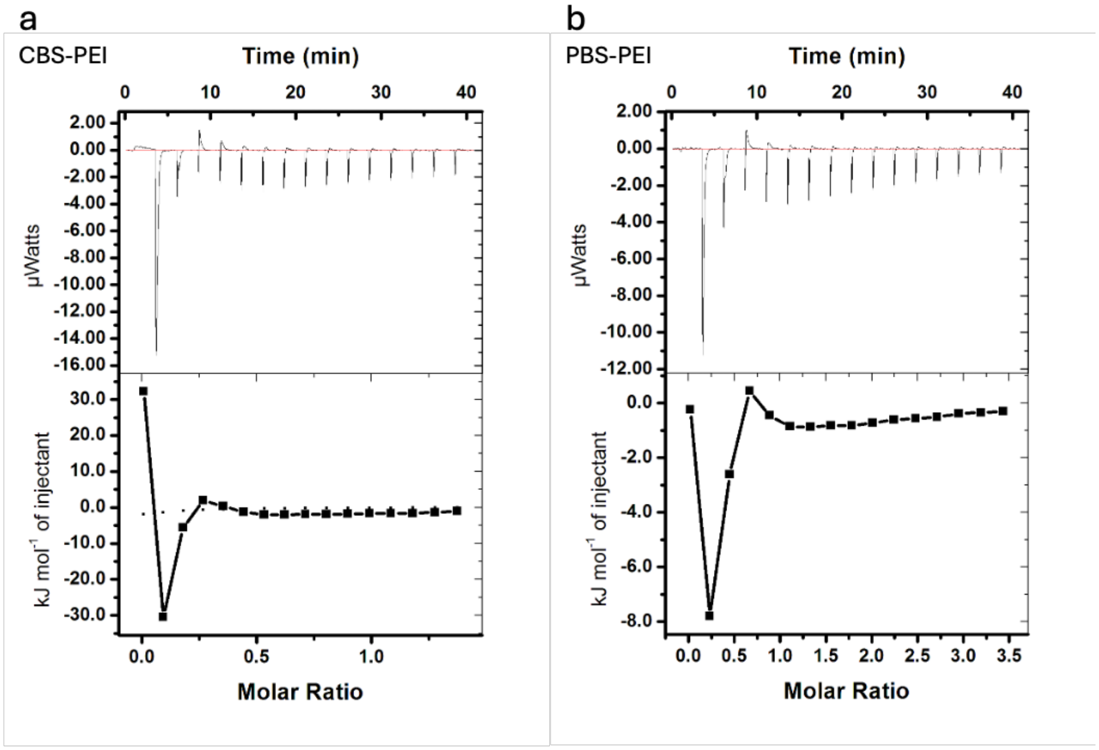

Supplementary Fig. 6. Isothermal titration calorimetry (ITC) analysis of buffer–PEI interactions. Representative ITC thermograms (upper panels) and corresponding integrated heat profiles (lower panels) for titration of **(a)** CBS and **(b)** PBS into PEI solution. Raw injection heat signals are shown as differential power (μW) versus time, and integrated heats are plotted as a function of the molar ratio of buffer ions to PEI. Citrate-containing buffer produces substantially larger heat changes compared to PBS under matched conditions, indicating stronger and more cooperative interactions between citrate ions and protonated PEI. Integrated heat profiles reflect the multivalent, non-stoichiometric nature of PEI–ion association.

2. The study focuses exclusively on citrate ions. To evaluate whether the observed effects are unique to trivalent citrate, control experiments with other multivalent ions, such as divalent (e.g., SO_4^{2-}) or alternative trivalent ions, should be included.

[Response]: We agree that control experiments with other multivalent anions strengthen the generality of our interpretation. In response, we evaluated PEI–pDNA assembly using Na_2SO_4 (a divalent anion) and sodium EDTA (an alternative multivalent anion) under matched NaCl and pH conditions as used in CBS. As shown in **Supplementary Fig. 2**, Na_2SO_4 produced less controlled growth kinetics and higher PDIs than citrate. In contrast, EDTA yielded a more linear growth profile and lower PDIs, qualitatively resembling citrate-mediated behavior.

This result is consistent with our report comparing phosphate and citrate. Phosphate acts as a monovalent and divalent control at pH 7 because it exists predominantly as $H_2PO_4^-$ and HPO_4^{2-}. The assembly results in sulfates are similar to those obtained in PBS. EDTA serves as a practical alternative high-valence anion at neutral pH, carrying 4 negative charges. While EDTA supports the generality of the multivalency-mediated effect, citrate remains particularly attractive for translational formulation due to its established biocompatibility and the absence of strong metal-chelation concerns associated with EDTA.

Together, these control experiments suggest that the effect we observe is not unique to citrate itself, but rather reflects a general principle: multivalent ions can reduce the effective charge density of PEI and influence the kinetics of complex formation. Among these, citrate provides a particularly practical combination of strong charge modulation, buffering capacity at neutral pH, and established biocompatibility, making it well suited for formulation and potential translational use.

We have added these results to the revised manuscript (**Supplementary Fig. 2**) and clarified this point in the Results section.

Page 7, line 191:

To examine whether the observed kinetic modulation is specific to citrate, we performed additional experiments using Na_2SO_4 (a divalent anion) and sodium EDTA (an alternative multivalent anion) under matched conditions (**Supplementary Fig. 2**). The divalent sulfate-containing buffer resulted in less controlled particle growth and higher PDIs. In contrast, the tetravalent EDTA exhibited more linear growth and lower PDIs than PBS, qualitatively resembling citrate-mediated behavior. While EDTA supports the generality of the multivalency-mediated effect, citrate remains particularly attractive for translational formulation due to its established biocompatibility and the absence of strong metal-chelation concerns associated with EDTA. These results collectively confirm that multivalent ions influence the kinetics of complex formation and particle growth by temporarily reducing the effective charge density of PEI.

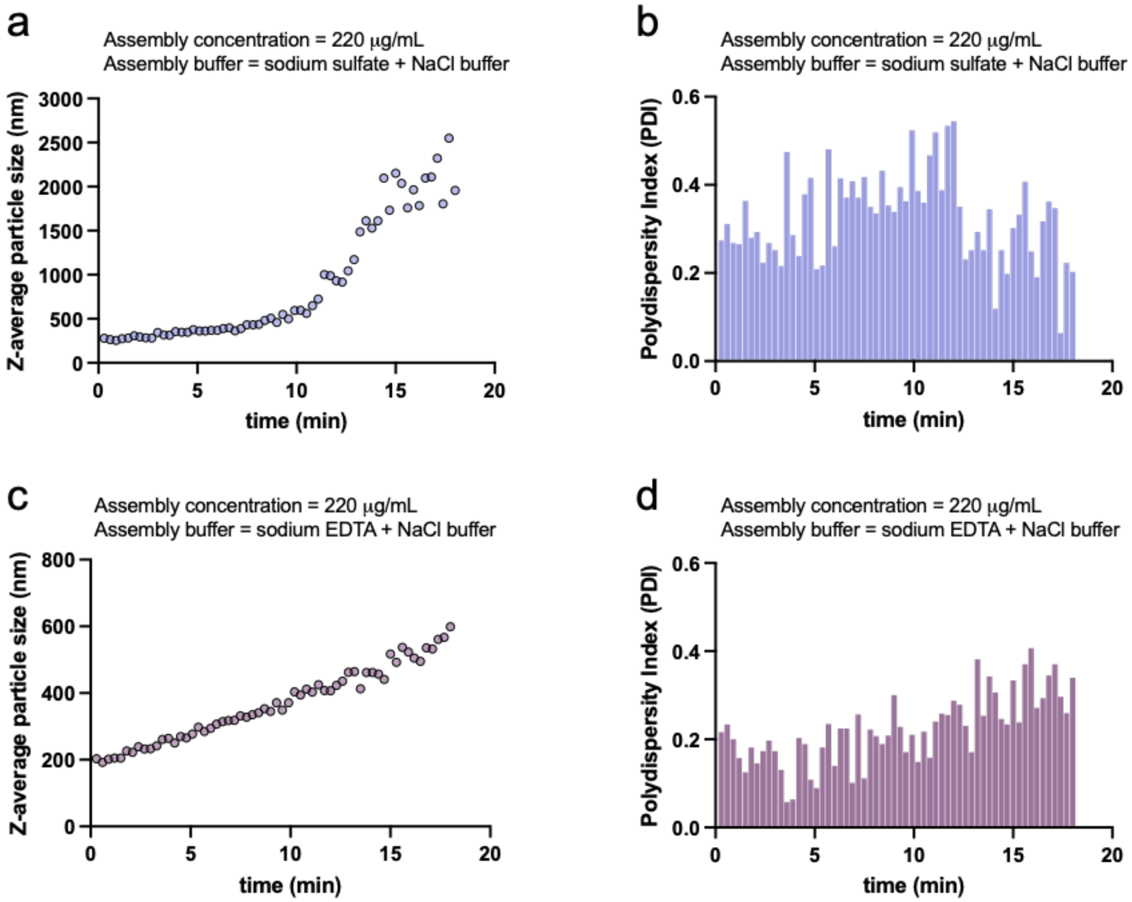

Supplementary Fig. 2. Effect of alternative multivalent anions on PEI–pDNA assembly kinetics. (a) Size evolution of PEI–pDNA particles assembled in Na_2SO_4-containing buffer (pH 7.0). **(b)** Corresponding polydispersity index (PDI) over time in Na_2SO_4 buffer. **(c)** Size evolution of PEI–pDNA particles assembled in sodium EDTA-containing buffer (pH 7.0). **(d)** Corresponding PDI over time in EDTA buffer. All measurements were performed by DLS immediately after mixing under identical formulation conditions.

3. The figure organization could be improved for clarity. It is recommended to re-group the panels to more clearly separate mechanistic studies (e.g., MD simulations), assembly kinetics, and functional outcomes (e.g., transfection efficiency).

[Response]: We thank the reviewer for the helpful suggestion regarding figure organization. In response, we revised Figure 3 to more clearly distinguish the different types of analyses presented in this section. The updated figure now explicitly highlights the experimental measurements, molecular dynamics simulations, and the proposed mechanistic interpretation, improving the readability of the mechanistic studies.

Regarding Figure 2i, although it reports transfection efficiency, we chose to retain it within Figure 2 because it directly evaluates the functional stability of the particles after freeze–thaw cycles. This measurement serves as an important validation of particle stability prior to the subsequent mechanistic and functional studies, and therefore forms part of the particle characterization section.

Figures 4 and 5 were not reorganized because they already represent distinct stages of the study. Figure 4 focuses on the scale-up experiments, while Figure 5 presents the functional outcomes. We believe this organization already clearly separates the assembly characterization, mechanistic investigation, scale-up validation, and biological performance of the system.

4. For Figures 2h and 2i, please use distinct bar colors or patterns to differentiate the experimental groups (e.g., PBS vs. CBS, different particle sizes). The current grayscale scheme may hinder readability.

[Response]: In response, we have revised Figures 2h and 2i so that the different conditions (t = 0 and t = 4 h; freshly prepared and freeze–thawed) are now distinguished using colors rather than symbols. The updated color-coded legend improves the clarity and allows the different groups to be more easily identified in the figure.

5. For Figure 2f (TEM images), please provide additional, in-focus TEM fields of view to better represent particle morphology and uniformity. The current images appear limited in scope and focus.

[Response]: We have included additional high-resolution, in-focus TEM images with other fields of view to better represent particle morphology and population uniformity. The revised figure now includes multiple representative regions for each condition, along with zoom-in views to clearly illustrate particle shape and structural features. These additional images confirm the reproducibility of the observed morphology and provide a more comprehensive visualization of particle uniformity under both PBS and CBS assembly conditions.

The updated TEM panels have been incorporated into the **Supplementary Fig. 3** to address this concern.

Page 8, line 207:

The pDNA/PEI particles assembled by PBS appeared to be randomly shaped agglomerates with drastically different sizes (**Fig. 2f, Supplementary Fig. 3a, b**), whereas the particles assembled by CBS are more uniform in size with a predominantly spherical shape (**Fig. 2g, Supplementary Fig. 3c, d**).

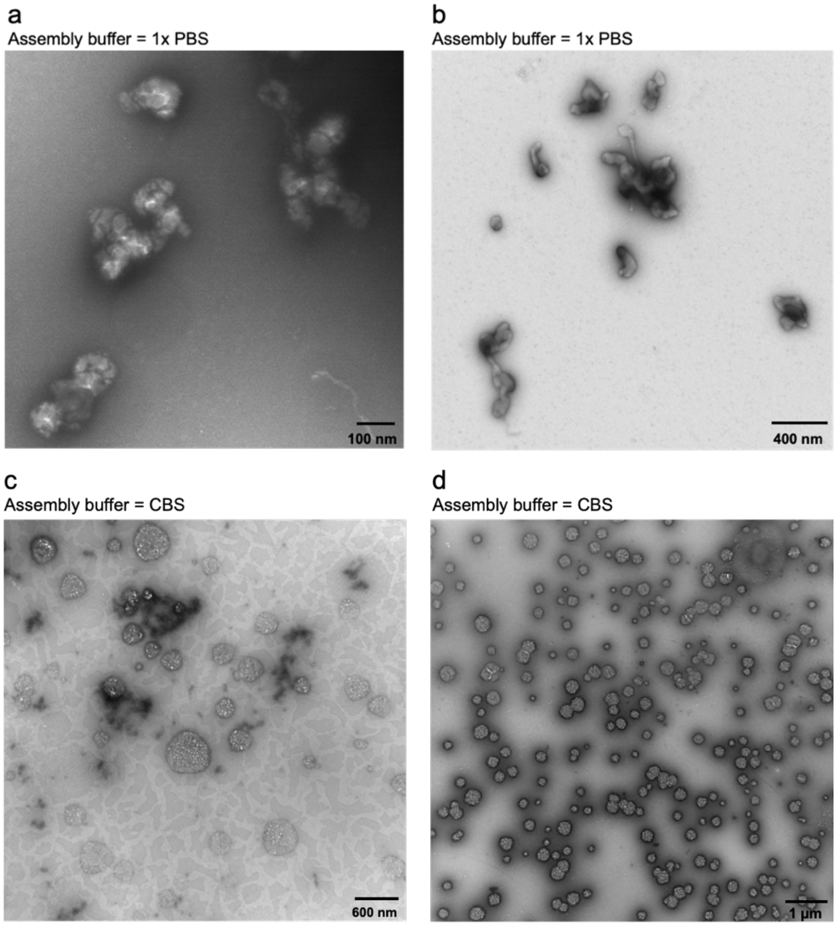

Supplementary Fig. 3. Transmission electron microscopy (TEM) characterization of PEI–pDNA particles assembled in PBS and CBS. Representative TEM images of particles assembled in PBS **(a, b)** and CBS (**c, d)** at two different magnifications. PBS-assembled particles exhibit more irregular and heterogeneous structures, whereas CBS-assembled particles display more spherical and uniform morphology. Scale bars are indicated in each panel.

6. Data on nanoparticle cytotoxicity is missing from the manuscript. This is a critical component for assessing the clinical translation potential of any novel transfection reagent. Standard cell viability assays should be performed.

[Response]: We agree with the reviewer and performed standard in vitro cell viability assays for the tested nanoparticle formulations under the same dosing conditions used in transfection experiments. The cells were stained with LIVE/DEAD Fixable Aqua Dead Cell Stain Kits (Thermo Fisher Scientific), and the results were analyzed with flow cytometry.

Across all tested formulations, cell viability remained comparable to the negative control group, with no statistically significant reduction observed between treatment groups (**Supplementary Fig. 8**), indicating that the observed differences in transfection performance are not confounded by cytotoxicity effects.

Page 12, line 354:

Importantly, cell viability assays showed no significant cytotoxicity across particle sizes compared with the negative control, at the dose used for transfection studies (**Supplementary Fig. 8**), indicating that the observed differences in transfection performance are not confounded by cytotoxicity effects.

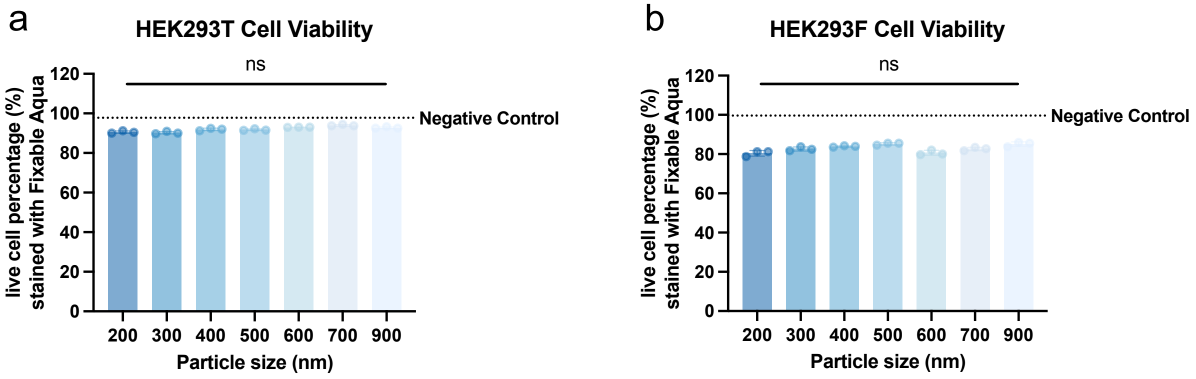

Supplementary Fig. 8. In vitro cytotoxicity assessment of PEI-pDNA particles. Cell viability was evaluated following treatment with indicated PEI-pDNA particles under the same dosing conditions used for transfection experiments on **(a)** HEK293T cells, **(b)** HEK293F cells. Viability was quantified using LIVE/DEAD Fixable Aqua staining, and the negative control is shown as a dotted line. No statistically significant reduction in cell viability was observed across groups. Data are presented as mean ± s.d. (n = 3).

7. The stability of the particles under physiologically relevant conditions is not evaluated. The particles should be incubated in cell culture media, both with and without 10% FBS, at 37°C. Changes in particle size (by DLS) and PDI should be monitored over a relevant time course. Serum protein adsorption can lead to aggregation or opsonization, which would severely limit practical application.

[Response]: We agree on the importance of stability under physiologically relevant conditions and the potential impact of serum protein adsorption. In response, we incubated 400-nm PEI–pDNA particles in cell culture medium (1:100 dilution as consistent with cell dosing condition) at 37 °C with and without 10% FBS, and monitored hydrodynamic size and PDI by DLS over time (**Supplementary Fig. 9**).

Across the monitored time course, we observed no evidence of aggregation (e.g., micron-scale growth or rapid precipitation) under either condition. In basal medium (no FBS), the particle size increased modestly, which we attribute to ionic-strength–dependent electrostatic screening that can promote mild inter-particle association. Importantly, this increase remained limited and did not progress to large aggregates within the 15-minute observation window.

In medium containing 10% FBS, the measured Z-average size decreased (to ~300 nm) with a moderate increase in PDI. We note that DLS in serum-containing media does not directly resolve protein corona formation; however, the observed size reduction is consistent with serum proteins providing steric stabilization that reduces weak inter-particle bridging and disperses loosely associated clusters, rather than inducing aggregation. Importantly, the intensity-weighted size

distribution shows that the dominant population remains in the submicron regime, with the principal peak still centered within the ~400–500 nm window and no emergence of large-aggregate peaks indicative of opsonization-driven clustering under these in vitro conditions.

Taken together, these data indicate that incubation in physiologically relevant media, both with and without serum, does not substantially compromise colloidal integrity at the dosing-relevant dilution and time scale. While serum adsorption is expected for non-PEGylated polyplex systems, our results suggest that such interactions are not destabilizing in this system and do not eliminate the particle size regime associated with the observed size-dependent transfection behavior.

We have added these results to the revised manuscript (**Supplementary Fig. 9**) and clarified this point in the Results section.

Page 12, line 357:

Additionally, particles incubated in cell culture medium with or without 10% FBS at 37 °C did not result in catastrophic aggregation over the relevant time window (**Supplementary Fig. 9**, next page).

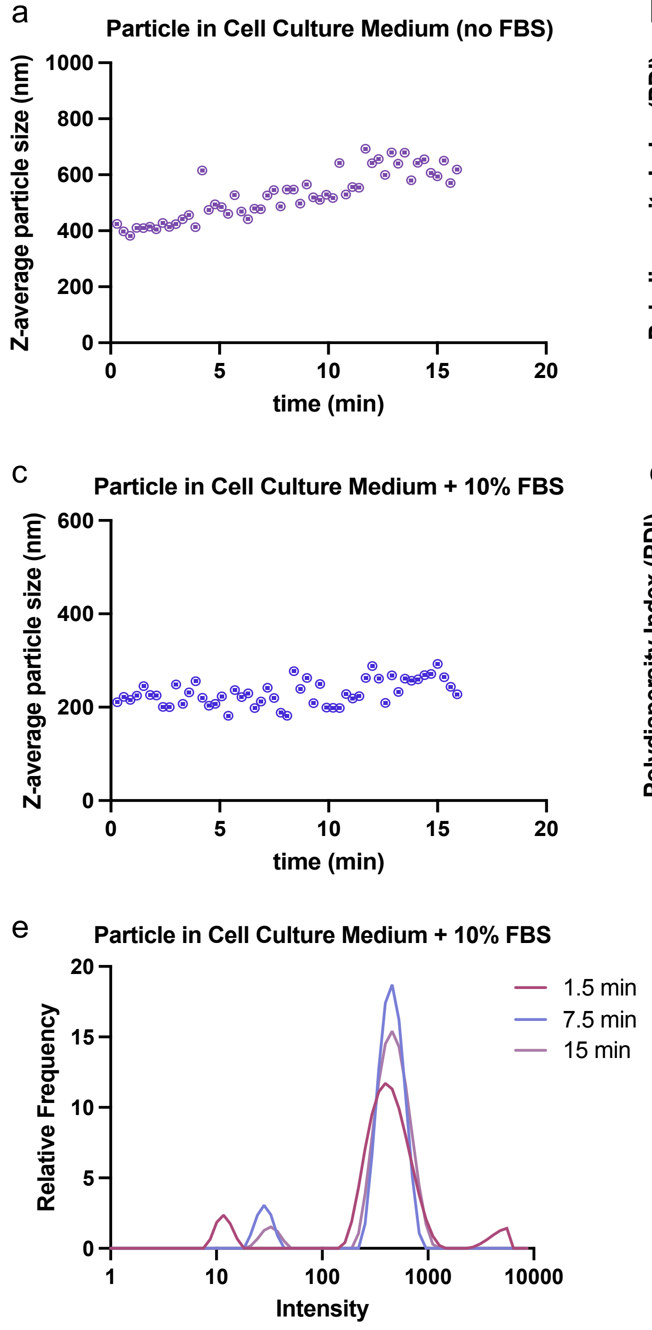

Supplementary Fig. 9. Size and polydispersity index (PDI) of ~400 nm PEI–pDNA particles incubated in cell culture medium at 37 °C, with and without 10% fetal bovine serum (FBS), measured by dynamic light scattering (DLS). Z-average hydrodynamic diameter of particles incubated in cell culture medium (**a**) without FBS and (**c**) with 10% FBS. Corresponding PDI values are shown for particles incubated (**b**) without FBS and (**d**) with 10% FBS. (**e**) Representative intensity-weighted size distribution of particles incubated in medium containing 10% FBS at early- (1.5 min), mid- (7.5 min) and late- (15 min) time point.
